# Efficiency and Quality of Generative AI–Assisted Radiograph Reporting

**DOI:** 10.1001/jamanetworkopen.2025.13921

**Published:** 2025-06-05

**Authors:** Jonathan Huang, Matthew T. Wittbrodt, Caitlin N. Teague, Eric Karl, Galal Galal, Michael Thompson, Ajay Chapa, Ming-Lun Chiu, Bradley Herynk, Richard Linchangco, Ali Serhal, J. Alex Heller, Samir F. Abboud, Mozziyar Etemadi

**Affiliations:** 1 Department of Radiology, Northwestern University Feinberg School of Medicine, Chicago, Illinois; 2 Research & Development, Northwestern Medicine Information Services, Chicago, Illinois; 3 Department of Biomedical Engineering, Northwestern University, Evanston, Illinois; 4 Department of Anesthesiology, Northwestern University Feinberg School of Medicine, Chicago, Illinois

## Abstract

**Importance:**

Diagnostic imaging interpretation involves distilling multimodal clinical information into text form, a task well-suited to augmentation by generative artificial intelligence (AI). However, to our knowledge, impacts of AI-based draft radiological reporting remain unstudied in clinical settings.

**Objective:**

To prospectively evaluate the association of radiologist use of a workflow-integrated generative model capable of providing draft radiological reports for plain radiographs across a tertiary health care system with documentation efficiency, the clinical accuracy and textual quality of final radiologist reports, and the model’s potential for detecting unexpected, clinically significant pneumothorax.

**Design, Setting, and Participants:**

This prospective cohort study was conducted from November 15, 2023, to April 24, 2024, at a tertiary care academic health system. The association between use of the generative model and radiologist documentation efficiency was evaluated for radiographs documented with model assistance compared with a baseline set of radiographs without model use, matched by study type (chest or nonchest). Peer review was performed on model-assisted interpretations. Flagging of pneumothorax requiring intervention was performed on radiographs prospectively.

**Main Outcomes and Measures:**

The primary outcomes were association of use of the generative model with radiologist documentation efficiency, assessed by difference in documentation time with and without model use using a linear mixed-effects model; for peer review of model-assisted reports, the difference in Likert-scale ratings using a cumulative-link mixed model; and for flagging pneumothorax requiring intervention, sensitivity and specificity.

**Results:**

A total of 23 960 radiographs (11 980 each with and without model use) were used to analyze documentation efficiency. Interpretations with model assistance (mean [SE], 159.8 [27.0] seconds) were faster than the baseline set of those without (mean [SE], 189.2 [36.2] seconds) (*P* = .02), representing a 15.5% documentation efficiency increase. Peer review of 800 studies showed no difference in clinical accuracy (χ^2^ = 0.68; *P* = .41) or textual quality (χ^2^ = 3.62; *P* = .06) between model-assisted interpretations and nonmodel interpretations. Moreover, the model flagged studies containing a clinically significant, unexpected pneumothorax with a sensitivity of 72.7% and specificity of 99.9% among 97 651 studies screened.

**Conclusions and Relevance:**

In this prospective cohort study of clinical use of a generative model for draft radiological reporting, model use was associated with improved radiologist documentation efficiency while maintaining clinical quality and demonstrated potential to detect studies containing a pneumothorax requiring immediate intervention. This study suggests the potential for radiologist and generative AI collaboration to improve clinical care delivery.

## Key Points

**Question:**

Is clinical use of artificial intelligence (AI)–generated draft radiograph reports associated with documentation efficiency, clinical accuracy, textual quality, and ability to promptly detect pneumothorax requiring intervention?

**Findings:**

In this cohort study, in 11 980 model-assisted radiograph interpretations in live clinical care, model use was associated with a 15.5% documentation efficiency improvement, with no change in radiologist-evaluated clinical accuracy or textual quality of reports. Of 97 651 radiographs analyzed for pneumothorax flagging, those containing clinically actionable pneumothorax were identified rapidly with high accuracy.

**Meaning:**

The findings suggest the potential for radiologist and generative AI collaboration to improve clinical care delivery.

## Teaser

This cohort study evaluates whether use of a generative artificial intelligence (AI) model for interpreting draft radiograph reports is associated with documentation efficiency, clinical accuracy, and ability to promptly detect pneumothorax requiring intervention.

## Introduction

Diagnostic imaging interpretation involves, in part, a multimodal distillation of clinical information from unstructured imaging into textual form. Advances in generative artificial intelligence (AI) methods bridging these modalities have the potential to accelerate the process of documenting clinical findings within medical images by radiologists.^1,2,3^ Considering increasing demand for radiological services^4^ and associated radiologist shortages worldwide,^5^ efficiency improvement through generative AI adoption is of great interest in broadening access to diagnostic imaging. Applicability of generative methods to modeling image-text relationships for plain radiograph studies has recently been established using a variety of adapted and bespoke vision-language models,^6,7,8,9,10,11,12,13,14^ with ever-improving outcomes on standard benchmarks.^15,16^ However, studies to date have focused on chest radiographs exclusively, and prospective clinical evaluations remain unpublished, to our knowledge.^3^

In this study, we considered 2 avenues for radiologist workflow augmentation by generative AI. First, AI-generated draft reports may facilitate more timely information consolidation.^10,11^ A sufficiently accurate AI draft can serve as a starting point for documentation so that the radiologist need not type or dictate from scratch or from a predefined template, much as an attending radiologist verifies and edits a trainee report. Second, an AI draft contains language remarking on the severity and chronicity of findings, enabling identification of studies warranting immediate radiologist attention more reliably than classification-based strategies.^17^ Of the immediately life-threatening pathologies reliably identifiable on radiography, pneumothorax is relatively common across clinical settings,^18^ making it a promising proof-of-concept target for generative AI-based prioritization.^19^

**Figure 1.**
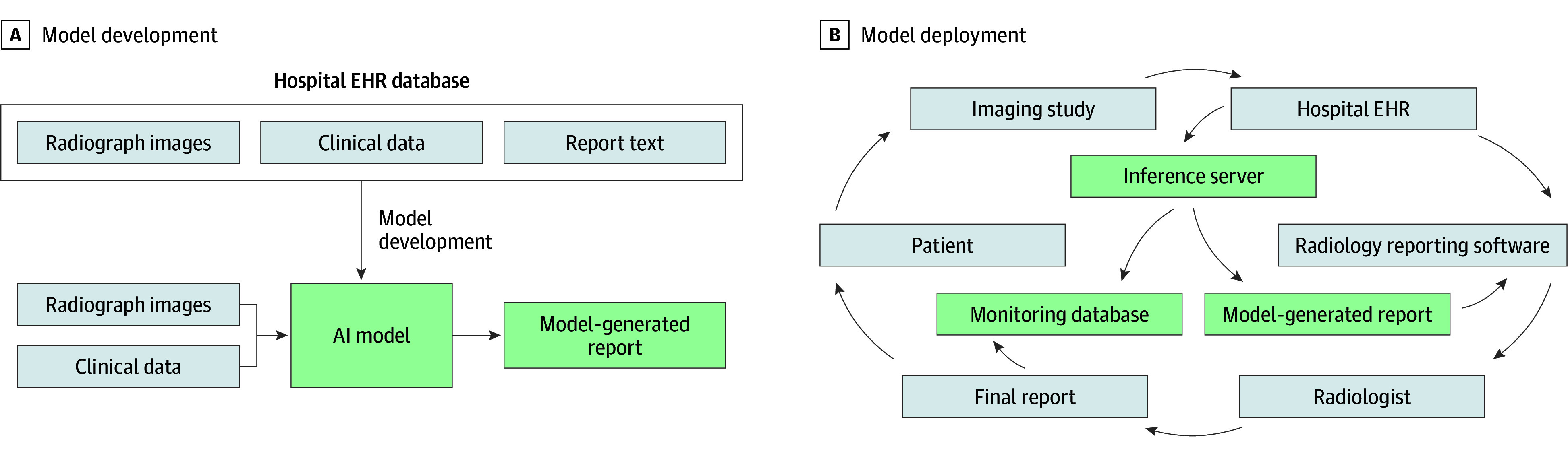
Overview of Model Development and Deployment A generative AI model capable of producing radiograph report text from input images and clinical data (reason for examination, procedure type, comparison information, and radiologist name) was developed using data from the electronic health record (EHR) of an academic hospital system. This model was then integrated into the live clinical workflow across the hospital system.

**Figure 2.**
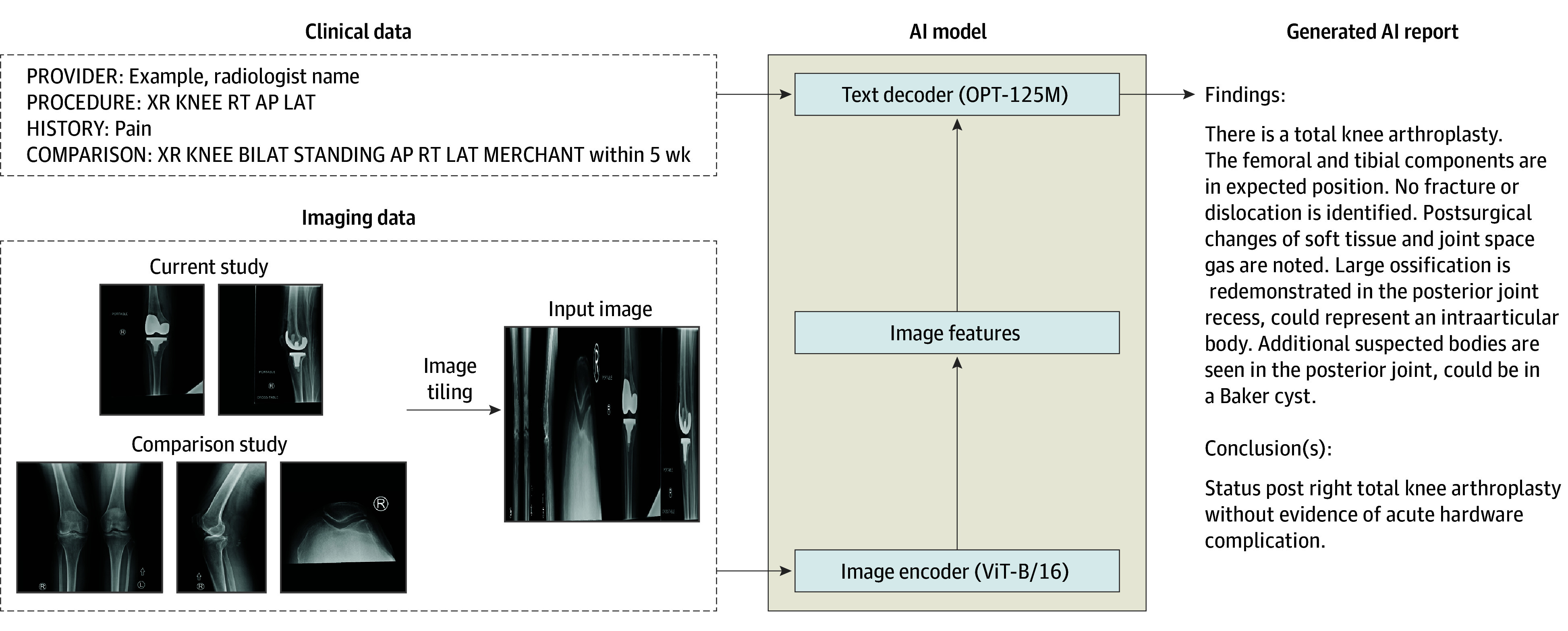
Model Design Illustration The model generates radiology reports given a plain radiograph study, a single comparison study, and basic clinical data. Study images are cropped to remove black, resized in the horizontal direction, and then tiled to a 1024 × 1024 resolution square with the current study images occupying the right half and comparison images on the left. The text decoder, a 125 million parameter Open Pretrained Transformer (OPT-125M) model, receives image features generated by the image encoder, a base-sized 86 million parameter vision transformer model with patch size 16 (ViT-B/16), and generates a report given a clinical data prompt that includes an interpreting radiologist name, the procedure name, the provided reason for examination, and the comparison procedure name and time interval. XR KNEE RT AP LAT indicates a 2-view radiograph of the right knee; XR KNEE BILAT STANDING AP RT LAT MERCHANT indicates a 3 view radiograph of the right knee.

We performed a prospective clinical evaluation (Figure 1) of these impacts of a generative AI model (Figure 2) capable of producing draft radiology reports for all plain radiographs, which was implemented within the live clinical workflow at our institution. We studied whether use of model-generated drafts was associated with radiologist documentation time and quantified the clinical accuracy and textual quality of final radiologist reports by peer review, comparing outcomes with baseline performance prior to model implementation. We also prospectively evaluated the accuracy of model-generated reports for flagging clinically significant, unexpected pneumothoraxes requiring physician intervention.

## Methods

### Model Inference and Deployment

The generative model used in this prospective cohort study was a multimodal encoder-decoder transformer-based model^20^ jointly conditioned on text and images, trained using an institutional dataset to produce free-text radiology reports (Figure 2). Architecture and training details are provided in eMethods 1 to 5 in Supplement 1. Model inference was seamlessly integrated with the institutional electronic health record (EHR) software (Epic; Epic Systems) and reporting software (PowerScribe; Nuance Communications), minimizing disruptions to established clinical routines (Figure 1 and eMethods 6 in Supplement 1). In the typical radiology workflow, imaging data from patients enters the EHR and is sent to radiology reporting software, used by radiologists to view imaging, review clinical history, and document interpretations (typically via voice dictation). These finalized reports are then used by other clinicians to guide clinical decision-making. In the model’s clinical integration, a server receives imaging and clinical data from the EHR as image acquisitions complete, performs inference to generate draft AI reports, and logs all activity to a monitoring database. The draft AI report is made available as custom fields included within a template selectable within the radiology reporting software as soon as inference completes, within seconds of image acquisition. Thus, radiologists may document reports by verifying and editing these AI-generated reports within their normal workflow. All model outputs, AI draft use, finalized reports, and documentation timing data are logged to the monitoring database. During the study period, the model was available via a PowerScribe template to a limited set of radiologists as part of a phased rollout by practice location and imaging section across the health system. Otherwise, radiologist workflows were unchanged. The study protocol was approved by the Western-Copernicus Group Institutional Review Board, with a waiver of informed consent given the minimal risk of data collection. The study followed the Strengthening the Reporting of Observational Studies in Epidemiology (STROBE) reporting guideline. Reporting was in accordance with the Checklist for Artificial Intelligence in Medical Imaging (CLAIM).^21^

### Study Population and Design

The study cohorts for this prospective cohort study were derived from radiographs obtained at our institution, a 12-hospital tertiary care academic health system, between November 15, 2023, and April 24, 2024, for which the model generated an AI draft report. To assess the association of model use with documentation efficiency, radiologists who interpreted at least 10 studies without trainee involvement using the model-generated draft were identified. A baseline dataset matched by chest or nonchest radiograph type was identified from the most recent consecutive studies interpreted by each radiologist without trainee involvement before their first model use. Radiologists without model use served as a control group, selected by randomly matching each model user to a radiologist within the same imaging section (eg, thoracic, emergency) matched by study count.

Studies for the peer review analysis (Box) were randomly sampled from the documentation efficiency dataset through March 14, 2024, with equal representation among radiologists following the power analysis described in eMethods 7 in Supplement 1. Raters were blinded to the model use status and reading radiologist for each study and did not review their own studies. The peer review platform is described in eMethods 8 in Supplement 1; eFigure 1 in Supplement 1 depicts the rating application.

Box. **Peer Review Rating Scales**

**Likert Scale for Clinical Quality**

1. Disagree with the majority of the report.
2. Disagree with critical findings; agree with noncritical findings.^a^
3. Agree with critical findings; disagree with noncritical findings.^a^
4. All findings are appropriately reported.

**Likert Scale for Text Quality**

1. Rewrite needed.
2. Minor wording or formatting changes needed (eg, grammar, organization).
3. Report uses appropriate word choice and formatting.

^a^
A critical finding was defined to be any finding that would change the immediate clinical management of the patient if reported incorrectly, in the radiologist’s judgment.

Flagging of clinically significant, unexpected pneumothorax used all studies analyzed by the prioritization system (detailed in eMethods 9 and eTable 1 in Supplement 1), which was live from February 5 to April 24, 2024, in a shadow deployment that ran in real time without surfacing alerts to clinicians. This system aimed to identify studies containing emergent pneumothoraxes in patients with low pretest probability (eg, excluding patients with recent thoracic surgery and small, clinically inconsequential pneumothoraxes).

### Statistical Analysis

To examine radiologist documentation time, a linear mixed-effects model (lme4 package in R, version 1.1-33 [R Project for Statistical Computing]) was fit to the data with repeated measures (fixed effects) of procedure type (nonchest, chest) and model use (before and after model implementation) with the random effect of radiologist. Significance testing for main effect estimates and interactions was completed using the car package in R, version 3.1-2. Only studies documented without trainee involvement (eg, drafting of a preliminary report by a resident physician) were considered for analysis. As a sensitivity analysis to investigate the influence of individual radiologists on the overall effect estimates, successive linear mixed-effects models were fit to the dataset, each excluding 1 radiologist. Secondary statistical analyses were performed to investigate factors associated with documentation time changes with model use (eMethods 10 in Supplement 1).

Likert scores between model and nonmodel reports were compared using a cumulative-link mixed model from the ordinal (version 2022.11-16) package in R fit with main effects of procedure type and model use with random effects of study and rater. Significance testing for main effect estimates and interactions was completed using the RVAideMemoire package in R, version 0.9-83. The signing radiologist was initially used as a covariate but was removed after not being significant. Where applicable, the Akaike information criterion and bayesian information criterion were used to determine model selection. For all analyses, if a significant main effect estimate was found, post hoc analyses were completed using the emmeans (version 1.8.6) package in R with Bonferroni-Holm corrections. The α level was set to *P* ≤ .05 to determine significance. All *P* values were 2-sided and are reported with Bonferroni-Holm correction where applicable. Data are presented as estimated marginal means with SE or effect estimates with margin of error. RadGraph,^22^ which extracts clinical entities and relations from chest radiograph reports, was used to quantify clinical information within reports as a proxy for report complexity and to calculate RadGraph F1^15^ scores between draft and edited reports. Word error rate was calculated using the torchmetrics module, version 1.4.2 (Lightning AI). Power analysis and subgroup analysis by pathology category were performed as described in eMethods 7 and 10, respectively, in Supplement 1. The proportions of reports with and without an addendum^23^ (identified using the PowerScribe database) were compared before and after model deployment using a χ^2^ test.

## Results

The datasets for study of documentation efficiency impact (23 960 radiograph studies from 14 460 unique patients), peer review (800 studies from 800 unique patients), and pneumothorax flagging (97 651 studies from 73 881 unique patients) were derived from 299 164 radiographs at our institution. Mean (SD) patient age for the documentation efficiency impact studies was 59.6 (17.5) years; 11 689 (48.8%) patients were female, 12 268 (51.2%) were male, and 3 (<0.1%) were other gender. For peer review studies, mean (SD) patient age was 57.5 (19.6) years; 457 patients (57.1%) were female, and 343 (42.9%) were male. For studies of pneumothorax flagging, mean (SD) patient age was 60.5 (18.1) years; 54 088 (55.4%) were female, 43 535 (44.6%) were male, 19 (<0.1%) were other gender, and 9 (<0.1%) had unknown gender. Demographic information is presented in eTable 2 in Supplement 1.

### Association With Radiologist Documentation Efficiency

Use of the model draft was associated with more efficient documentation. Of the 11 980 studies interpreted with the model, 9791 (81.7%) were chest and 2189 (18.3%) were nonchest radiographs. The distribution of included radiographs is detailed in eFigure 2 and eTable 3 in Supplement 1. Inference completed in a median of 3 seconds (IQR, 2-4 seconds). The chest radiographs were interpreted by 12 radiologists reading a median of 202 studies (IQR, 49-938 studies) and the nonchest radiographs by 15 radiologists reading a median of 60 studies (IQR, 28-122 studies) (eTable 4 in Supplement 1). The premodel matched set comprised 11 980 studies mirroring the model use set in chest and nonchest composition and radiologist representation. No significant differences in anatomy representation were observed between the model use and premodel sets. The median word error rate of model-generated compared with final reports, measured as the ratio of substitutions, additions, and deletions to generated word count, was 0.31 (IQR, 0.16-0.60) for chest and 0.63 (IQR, 0.40-0.85) for nonchest studies. Examples of edited model reports are given in eTable 5 in Supplement 1.

**Figure 3.**
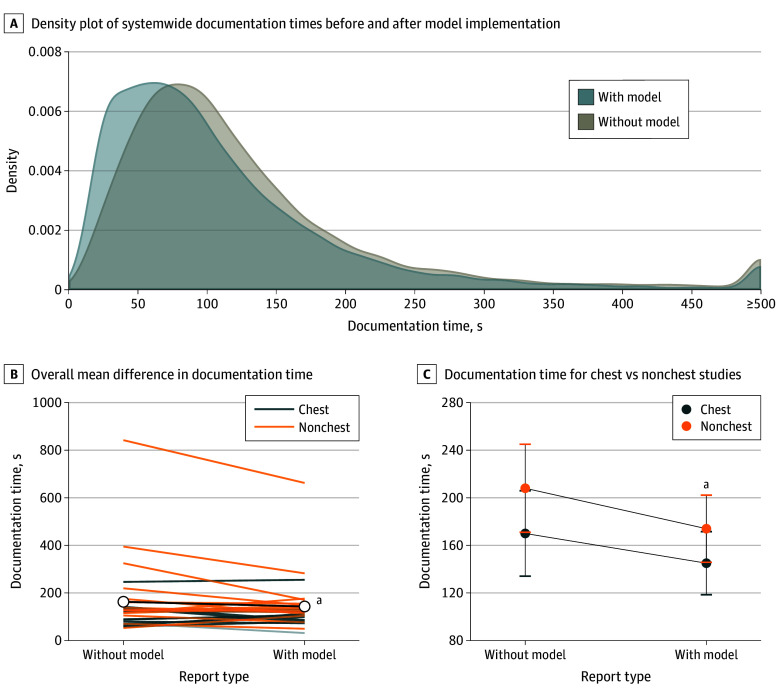
Radiologist Documentation Times for Interpretations With and Without the Model B, Black line indicates mean difference in documentation time with vs without the model draft. Other lines indicate individual radiologist differences. C, Whiskers indicate SEs. ^a^Association between model use and documentation time was significant (*P* = .02).

There was a significant association between model use and documentation time (χ^2^ = 5.36; *P* = .02), with model-assisted documentation times (mean [SE] of 159.8 [27.0] seconds) being significantly faster than for nonmodel studies (mean [SE] of 189.2 [36.2] seconds) by a mean of 29.4 (margin of error [ME], 21.5) seconds (*z* = 2.29; *P* = .02), corresponding to a 15.5% increase in per-study documentation efficiency (Figure 3). There was also a significant association between procedure type (χ^2^ = 20.98; *P* < .001) and documentation time, with documentation time for nonchest studies being significantly greater (by a mean [ME] of 33.3 [14.1] seconds; *z* = 4.63; *P* < .001) than for chest studies. The procedure type by model interaction was not significant (χ^2^ = 0.64; *P* = .43), indicating that no evidence of the procedure type modifying the association of model use with documentation time was found. In the control group comprising 10 897 studies each before and after model implementation, this analysis found no evidence for change in documentation efficiency for radiologists with vs without model use (eAppendix 1 in Supplement 1).

Documentation times across pathology and anatomy subgroups are detailed in eFigure 3 in Supplement 1, highlighting efficiency benefits of model use across a wide range of clinical abnormalities. Moreover, in the sensitivity analysis, all splits showed a significant association between model use and improved documentation time, with median documentation time improvement of 30.4 seconds (IQR, 28.3-31.5 seconds) with model use. Thus, removing 1 radiologist did not alter the overall association of AI model use with radiologist documentation time. Results from analysis of factors associated with documentation efficiency gain with model use are presented in eAppendix 2 in Supplement 1.

In addition, as a measure of documentation quality, we investigated the rate at which addenda used to rectify reporting errors were made to reports before and after model implementation. In the 11 980 premodel reports, addenda were made in 16 (0.13%), while in the 11 980 model-assisted reports, addenda were made in 17 (0.14%) (χ^2^ = 0.03; *P* = .86), suggesting unchanged radiograph interpretation quality.

### Peer Review of Model-Assisted Reports

The peer review analysis included 2 sets (chest and nonchest) of 400 studies, each comprising 200 premodel studies and 200 model use studies. Regression output tables are provided in eTable 6 in Supplement 1 and information regarding raters in eAppendix 3 in Supplement 1.

**Figure 4.**
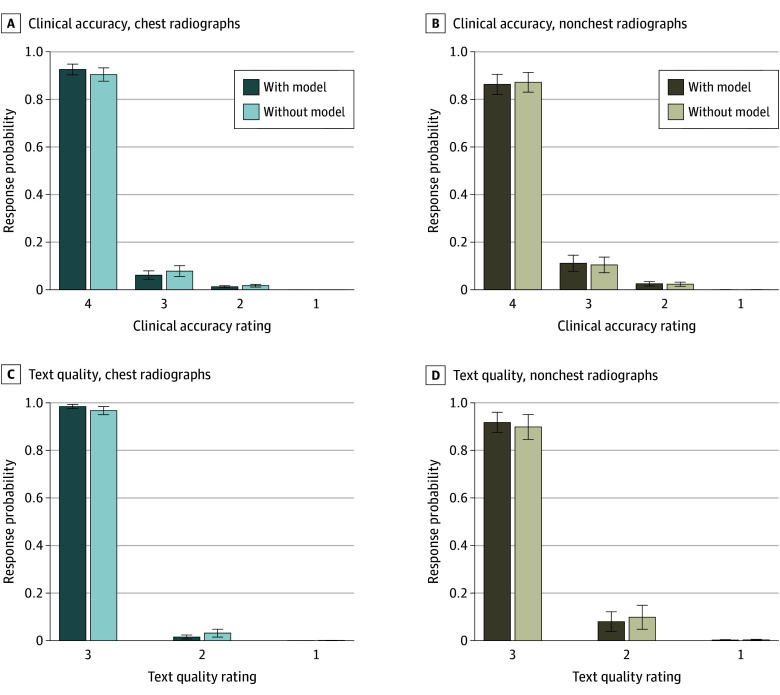
Distribution of Peer-Review Evaluation Scores for Radiograph Interpretations With and Without the Model

Regarding clinical accuracy (Figure 4 and Box), there was no association between model use and clinical accuracy (χ^2^ = 0.68; *P* = .41), indicating that there was no difference in clinical quality of reports documented with or without the model. There was a significant association with study type (χ^2^ = 11.54; *P* < .001), with post hoc tests revealing that chest studies were rated higher than nonchest studies by a mean (ME) of 0.65 (0.37) points on a scale of 1 to 4, with 4 indicating all findings were appropriately reported (*z* = 3.38; *P* < .001). There was no interaction between model use and study type (χ^2^ = 1.75; *P* = .19). The proportion of studies with unanimous agreement was comparable to a previously reported value^24^ at 61.4% (chest, 64.5%; nonchest, 58.2%), with a Kendall *W* of 0.37 (n = 4 raters; χ^2^ = 321.82; *df* = 220; *P* < .001) and 0.41 (n = 4 raters; χ^2^ = 159.42; *df* = 98; *P* < .01) for chest and nonchest studies, respectively, indicating fair agreement among raters.

On secondary analysis, model-assisted and nonmodel reports did not differ by error type (context, extraneous content, or omission) identified by reviewing radiologists. No model use–by-procedure type interactions were observed for any error type. Moreover, clinical accuracy scores did not differ significantly between ratings of model and nonmodel studies for any pathology category. The pathology distribution is given in eFigure 2 in Supplement 1.

Regarding textual quality (Figure 4 and Box), there was no association with model use (χ^2^ = 3.62; *P* = .06), indicating no difference in textual quality in reports documented with and without model use. Full cumulative-link mixed model outputs are described in eAppendix 4 in Supplement 1. The proportion of studies with unanimous agreement of text scores was 76.8% (chest, 83.5%; nonchest, 70.0%), with a Kendall *W* of 0.29 (n = 4 raters; χ^2^ = 250.10; *df* = 219; *P* = .07) and 0.34 (n = 4 raters; χ^2^ = 130.10; *df* = 98; *P* = .01) for chest and nonchest studies, respectively, indicating fair agreement among raters.

### Flagging Clinically Significant, Unexpected Pneumothorax

During the prioritization system shadow deployment period, 97 651 studies for which the model generated a report were screened (eFigures 4 and 6 in Supplement 1). Of these, 78 were flagged in real time by the prioritization system as containing a pneumothorax warranting immediate attention; 56 (71.8%) were true pneumothoraxes when cross-referenced with the final interpreting radiologist’s report. Furthermore, 30 (38.5%) resulted in calls to the clinical team ordering the imaging study. Priority flags were available in a median of 24.0 seconds (IQR, 21.3-44.8 seconds) after study completion, while radiologist notifications took place at a median of 24.5 minutes (IQR, 14.6-56.0 minutes).

On retrospective examination of the 97 651 final radiologist-documented reports, 33 studies contained a pneumothorax, resulted in clinical team notification, and met prioritization criteria, of which 24 (72.7%) had been flagged by the aforementioned live prioritization system. Thus, the prioritization system had a sensitivity of 72.7% and specificity of 99.9% for detection of unexpected pneumothoraxes warranting clinical team notification. Of the remaining 9 studies not flagged by the system (27.3%), all but 1 were qualified as “small,” “suspected,” or “uncertain” by the radiologist, whereas the model-generated report stated that there was no significant pneumothorax. Of note, 6 studies (20.0%) had been flagged by the system and resulted in calls to the clinical team but did not meet prioritization criteria on retrospective examination; this was due to delayed availability of patient location information and prior imaging interpretations in the EHR.

### Evaluation by Automated Metrics of Radiograph Quality

Model performance was benchmarked on internal and external test sets using automated metrics, demonstrating performance comparable to the recent state of the art (eTable 7 in Supplement 1). Ablation and scaling studies (eAppendix 5 and eFigure 5 in Supplement 1) demonstrated the utility of the tiling and clinical prompting as well as the potential value of increasing model size.

## Discussion

This study described, for the first time to our knowledge, prospective evaluation of a generative AI model for imaging interpretation in a live radiology clinical practice setting. We found a 15.5% documentation efficiency benefit with no decrease in clinical accuracy on peer review, representing a net time savings of over 63 documentation hours over the study period, or a reduction from roughly 79 to 67 radiologist shifts required to provide coverage. More efficient radiologist documentation may alleviate shortfalls in imaging access^5^ while reducing burnout.^25^ Notably, integration of new tools warrants careful attention to minimize workflow fragmentation or alert fatigue.^26^ In this study, an AI model was seamlessly integrated into an existing radiology workflow and mirrored the established clinical practice of editing trainee-produced draft reports, maximizing potential clinician benefit.

Most studies examining AI-assisted radiograph interpretation in preclinical^27,28,29^ and clinical^30^ settings have used classification-based models, which provide disjoint outputs less applicable to the holistic review that underlies report documentation. While studies have demonstrated accuracy benefits of radiologist-AI collaboration, particularly for less experienced clinicians,^27,28,29,30^ substantial heterogeneity in response has been recently described.^31^ Nonetheless, assistance by generative models in particular remains understudied. A recent study found radiologist preference for radiologist reports over edited AI reports on the MIMIC-CXR dataset^32^ but not an internal dataset, highlighting that both model error and clinical practice differences contribute to clinician disagreement.^9^ Further study of AI collaboration in clinical settings is needed to inform continued optimization of clinical deployments.

We also provided a proof-of-concept framework for extension of draft AI reporting to prioritization of critical studies, demonstrating high sensitivity and specificity for detection of clinically actionable pneumothorax. Although most flagged pneumothoraxes were noted by radiologists within 30 minutes, the system identified several preventable cases of delayed care. Notable examples included a patient with a large pneumothorax who was discharged from the emergency department based on a preliminary interpretation that missed this finding and then was called back 6 hours later after an attending radiologist’s overread and another patient who was undergoing inpatient workup for pneumonia who had a radiographically evident pneumothorax that was only noted by the care team following an acute oxygen desaturation event 11 hours after imaging acquisition.

Existing commercially available systems to identify pneumothoraxes on radiography use classification methods directly on imaging data, achieving sensitivities ranging from 63% to 90% and specificities ranging from 98% to 100%.^19,33,34^ However, they fail to consider relevant clinical context in report text reflecting the necessity of intervention, such as severity and chest tube presence, which may lead to extraneous alerts for cases of known or clinically insignificant pneumothorax. In this study, analysis of model-generated report text enabled the prioritization system to produce just over 1 alert per day, showing the potential of generative AI–based prioritization to safeguard against delayed care while minimizing alert fatigue. Further evaluation of performance and extension to other clinical findings may lay the groundwork for regulatory approval and broader adoption.

To date, generative radiograph models have exclusively studied thoracic pathology,^6,7,8,9,10,11,12,13^ while our model produces reports for radiographs covering all anatomy. Documentation efficiency in this study improved for chest and musculoskeletal radiographs despite the relatively higher word error rate for nonchest reports, evidencing the clinical utility of the AI model throughout radiograph modalities. Considering established challenges of quantifying report text quality^15^ and a lack of datasets pairing musculoskeletal radiographs with reports,^35,36,37^ continued development and evaluation of generative models tailored to musculoskeletal radiography will rely on efforts to translate datasets and metrics available for chest radiographs while determining modeling practices that best account for differences between the modalities.

### Limitations

This study has limitations. Although our institution serves a diverse patient population, the radiographs and radiologists studied may not be representative of other populations. Additionally, the repeated-measures study design used radiologists as their own controls because direct comparison between an edited AI draft and an independently documented draft was not possible; a study design involving double reading of radiographs may mitigate this. Continued longitudinal study of model use is needed to characterize potential performance drift and investigate the translation of per-study efficiency gains to longer-term productivity changes and factors such as burnout. Further incorporation of clinical context and extended comparison studies may improve model performance. Finally, due to this study’s nonrandomized nature, further experimental evidence is needed to build on its preliminary findings to establish generalizable results regarding draft reporting by AI.

## Conclusions

In this prospective cohort study of clinical use of a generative model for draft radiological reporting, model use was associated with improved radiologist documentation efficiency while maintaining clinical quality and, moreover, demonstrated potential to detect studies containing a pneumothorax requiring immediate intervention. Our results provide initial evidence for benefits of draft reporting using generative AI tools and a framework by which clinician-AI collaboration may effectively integrate into and improve existing clinical workflows.

## Notes

Supplement 1. **eMethods 1.** Model Development Dataset **eMethods 2.** Model Architecture **eMethods 3.** Model Training **eMethods 4.** Model Evaluation Using Automated Metrics **eMethods 5.** Ablation and Model Scaling Evaluation **eMethods 6.** Model Implementation and Use **eMethods 7.** Power Analysis for Peer Review Study **eMethods 8.** Peer Review Evaluation Scale and Platform **eMethods 9.** Pneumothorax Prioritization Strategy **eMethods 10.** Secondary Statistical Analyses **eAppendix 1.** Control Group Radiologist Documentation Efficiency **eAppendix 2.** Factors Associated With Documentation Efficiency Improvement **eAppendix 3.** Peer Review Radiologist Information **eAppendix 4.** Cumulative Link Mixed Model Outputs for Textual Quality Peer Review **eAppendix 5.** Evaluation by Automated Metrics of Radiograph Quality **eFigure 1.** Peer Review Web Application **eFigure 2.** Model Performance Across Pathology Subgroups **eFigure 3.** Documentation Time Change by Radiograph Subgroup **eFigure 4.** Pneumothorax Flag Criteria **eFigure 5.** Ablation Studies and Model Scaling Investigation **eFigure 6.** Flowchart for Pneumothorax-Flagging Study Inclusion **eTable 1.** Prioritization Text Search Exclusions **eTable 2.** Study Demographic Information **eTable 3.** Training and Evaluation Set Radiograph Breakdown by Anatomy **eTable 4.** Timing Data per Radiologist **eTable 5.** Example Model-Generated Reports and Radiologist Edits **eTable 6.** Regression Outputs for Mixed-Effects Models **eTable 7.** Evaluation Using Automated Metrics
eReferences

Supplement 2.
Data Sharing Statement
