# Supplementary material for: Efficiency and Quality of Generative AI–Assisted Radiograph Reporting
Source: JAMA Netw Open. 2025 Jun 5;8(6):e2513921. doi: 10.1001/jamanetworkopen.2025.13921 (PMC12142447; doi:10.1001/jamanetworkopen.2025.13921)
Supplement: Supplement 2. — Data Sharing Statement [file jamanetwopen-e2513921-s002.pdf]

Data Sharing Statement

Huang. Efficiency and Quality of Generative AI–Assisted Radiograph Reporting. *JAMA Netw Open*. Published June 05, 2025. doi:10.1001/jamanetworkopen.2025.13921

Data
Data available: No

Additional Information
Explanation for why data not available: The clinical dataset of radiograph studies and reports were collected from the Northwestern Medicine health system electronic health record and cannot be made publicly available as they contain protected health information. The MIMIC-CXR dataset used for model evaluation is available at https://physionet.org/content/mimic-cxr/2.0.0/.
