# Supplementary material for: Efficiency and Quality of Generative AI–Assisted Radiograph Reporting
Source: JAMA Netw Open. 2025 Jun 5;8(6):e2513921. doi: 10.1001/jamanetworkopen.2025.13921 (PMC12142447; doi:10.1001/jamanetworkopen.2025.13921)
Supplement: Supplement 1. — eMethods 1. Model Development Dataset eMethods 2. Model Architecture eMethods 3. Model Training eMethods 4. Model Evaluation Using Automated Metrics eMethods 5. Ablation and Model Scaling Evaluation eMethods 6. Model Implementation and Use eMethods 7. Power Analysis for Peer Review Study eMethods 8. Peer Review Evaluation Scale and Platform eMethods 9. Pneumothorax Prioritization Strategy eMethods 10. Secondary Statistical Analyses eAppendix 1. Control Group Radiologist Documentation Efficiency eAppendix 2. Factors Associated With Documentation Efficiency Improvement eAppendix 3. Peer Review Radiologist Information eAppendix 4. Cumulative Link Mixed Model Outputs for Textual Quality Peer Review eAppendix 5. Evaluation by Automated Metrics of Radiograph Quality eFigure 1. Peer Review Web Application eFigure 2. Model Performance Across Pathology Subgroups eFigure 3. Documentation Time Change by Radiograph Subgroup eFigure 4. Pneumothorax Flag Criteria eFigure 5. Ablation Studies and Model Scaling Investigation eFigure 6. Flowchart for Pneumothorax-Flagging Study Inclusion eTable 1. Prioritization Text Search Exclusions eTable 2. Study Demographic Information eTable 3. Training and Evaluation Set Radiograph Breakdown by Anatomy eTable 4. Timing Data per Radiologist eTable 5. Example Model-Generated Reports and Radiologist Edits eTable 6. Regression Outputs for Mixed-Effects Models eTable 7. Evaluation Using Automated Metrics eReferences [file jamanetwopen-e2513921-s001.pdf]

Supplementary Online Content

Huang J, Wittbrodt M, Teague CN, et al. Efficiency and quality of generative AI-assisted radiograph reporting. *JAMA Netw Open*. 2025;8(6):e2513921. doi:10.1001/jamanetworkopen.2025.13921

eMethods 1. Model Development Dataset
eMethods 2. Model Architecture
eMethods 3. Model Training
eMethods 4. Model Evaluation Using Automated Metrics
eMethods 5. Ablation and Model Scaling Evaluation
eMethods 6. Model Implementation and Use
eMethods 7. Power Analysis for Peer Review Study
eMethods 8. Peer Review Evaluation Scale and Platform
eMethods 9. Pneumothorax Prioritization Strategy
eMethods 10. Secondary Statistical Analyses
eAppendix 1. Control Group Radiologist Documentation Efficiency
eAppendix 2. Factors Associated With Documentation Efficiency Improvement
eAppendix 3. Peer Review Radiologist Information
eAppendix 4. Cumulative Link Mixed Model Outputs for Textual Quality Peer Review
eAppendix 5. Evaluation by Automated Metrics of Radiograph Quality
eFigure 1. Peer Review Web Application
eFigure 2. Model Performance Across Pathology Subgroups
eFigure 3. Documentation Time Change by Radiograph Subgroup
eFigure 4. Pneumothorax Flag Criteria
eFigure 5. Ablation Studies and Model Scaling Investigation
eFigure 6. Flowchart for Pneumothorax-Flagging Study Inclusion
eTable 1. Prioritization Text Search Exclusions
eTable 2. Study Demographic Information
eTable 3. Training and Evaluation Set Radiograph Breakdown by Anatomy
eTable 4. Timing Data per Radiologist
eTable 5. Example Model-Generated Reports and Radiologist Edits
eTable 6. Regression Outputs for Mixed-Effects Models
eTable 7. Evaluation Using Automated Metrics
eReferences

This supplementary material has been provided by the authors to give readers additional information about their work.

© 2025 Huang J et al. *JAMA Network Open*.

eMethods 1: Model development dataset

Model development was performed using 5,920,184 institutional plain film radiography studies taken from May 1999 through June 2022. All radiograph modalities and acquisition types (e.g. portable, fixed, etc.) were included. A patient-level 70/20/10 train/validation/test split yielded a training set of 4,186,288 studies, a validation set of 1,147,172 studies, and a test set of 425,877 studies. A breakdown of the training set studies by anatomy type is given in eTable 4.

A structured clinical information prompt was generated from available clinical information associated with each study by querying the electronic health record database via structured query language scripts. First, the “PROVIDERS:” field is populated with all radiologist names associated with interpretation of the study, separated by semicolons; the “PROCEDURE:” field is populated with the name of the imaging procedure performed; and the “HISTORY:” field is populated with the reason for exam documented as part of the imaging study order. Any information that could not be identified was listed as “Unspecified”. Next, the “COMPARISON:” field is populated with the name of the most recent prior radiograph study of the same body part available in the database, as well as the time interval (“within 4 hours”, “within 24 hours”, “within 1 week”, or “within X weeks”, with X denoting the number of weeks) between the two studies. Else, if no comparison was identified, the field was populated as “None”. Finally, a special token, “<_begin_report_>” is added to denote the end of the clinical information prompt, after which the report body is to be generated.

© 2025 Huang J et al. *JAMA Network Open*.

eMethods 2: Model architecture

The generative model is a multimodal encoder-decoder transformer-based model[1] jointly conditioned on text and images, trained to produce free text radiology reports. Weights for the base-size vision transformer[2] used as the vision encoder were initialized from a model pre-trained on ImageNet in a self-supervised manner using DINO[3] with a patch size of 16 (https://huggingface.co/facebook/dino-vitb16). As radiographs are monochrome, requiring only one color channel, the patch embeddings of this model were combined to a single channel by summing the weights across the channel dimension.

Text decoder weights were initialized from the 125 million parameter Open Pre-trained Transformer (OPT)[4] checkpoint (https://huggingface.co/facebook/opt-125m). The decoder tokenizer was based on the default RoBERTa[5] tokenizer trained from scratch using the standard byte-pair encoding algorithm[6] with a 5,000 token vocabulary size on the training dataset, with replacement of spaces with a custom string so that splitting was not performed on spaces, enabling encoding of commonly used phrases containing multiple words into a single token. Finally, the special "<_begin_report_>" token was added to the vocabulary. To accommodate this custom tokenizer, the OPT token embeddings layer was re-initialized to size 5,001 using Kaiming Initialization.[7] Cross-attention layers were initialized from scratch using Kaiming Initialization. Decoder position encodings were truncated to length 512.

© 2025 Huang J et al. *JAMA Network Open*.

eMethods 3: Model training

Encoder position embeddings were interpolated to enable fine-tuning at the 1024 x 1024 input resolution. The left half of this input image is reserved for the comparison study (or set to black if none exists), and the right for the current study. To accommodate multi-image studies, all images in a study underwent a rotation step to horizontal alignment, rectangular cropping of background black pixels, scaling to the full 16-bit range, and resizing to height of 1024 pixels. All images for a study were then tiled horizontally in a random order, then horizontally resized to 512 pixels and placed in the appropriate half of the input image. The order of tiling was randomized by the data loader as a data augmentation step.

During training and inference, the tokenized clinical information prompt was provided to the text decoder directly as a prompt from which generation proceeds. In training, a custom mask was also provided to prevent the loss function from considering the clinical information prompt, so that the only learning signal was derived from the report body.

The model was trained for 20 epochs on a machine with 4 80GB NVIDIA A100 graphics processing units (GPUs) to optimize the image captioning loss for report findings prediction conditioned on the input image and clinical information prompt. A learning rate of 2.75E-4, warmup ratio of 0.025, and global batch size of 96 were used with Adam optimization with parameters β_1 of 0.9 and β_1 of 0.999.

© 2025 Huang J et al. *JAMA Network Open*.

eMethods 4: Model evaluation using automated metrics

To enable subgroup analysis of changes in documentation time with model usage by pathology, the CheXbert labeler[8] was applied to identify pathologies present in final radiologist-documented report text, taking "positive" outputs to indicate pathology presence and "negative", "uncertain", and "blank" outputs to indicate pathology absence. An additional "Finding" category was included to indicate presence of any radiographic finding by taking the inverse of the "No finding" label. For non-chest studies, only the subgroups of "Fracture", "Support devices", "No finding", and "Finding" were used, as these pertain to non-chest radiographs. Subgroups by radiograph anatomy were additionally investigated. Data are presented as means and 95% confidence intervals were computed using 500 bootstrap samples.

To evaluate model performance, 5,000 chest and 5,000 non-chest studies were randomly sampled from the held-out test dataset. Clinically-aligned metrics (CheXbert vector score, RadGraph F1, and RadCliQ-v0) were used to evaluate quality of model-generated reports compared to the ground truth reports using a standard chest radiograph report evaluation package. [9] To prospectively evaluate model accuracy, 5,000 chest and 5,000 non-chest studies were randomly sampled from the set of radiographs interpreted without model usage from November 15, 2023 to April 24, 2024 and evaluated in the same way. Reports were generated using the same prompting scheme as in the live clinical deployment.

To evaluate model performance across pathology subgroups, the CheXbert labeler was applied as described above to calculate pathology-specific F1 scores as well as micro- and macro-F1 scores by comparing CheXbert labels of model outputs to those in final radiologist reports for the prospective quality evaluation dataset, the entire prospective model-assisted dataset, and the prospective test dataset. Data are presented as means and 95% confidence intervals were computed using 500 bootstrap samples.

Moreover, generalization to external datasets was examined by benchmarking model performance on MIMIC-CXR, [10] a dataset of chest radiographs widely used for development and evaluation of report generation models. Both zero-shot and fine-tuned model evaluation was performed on the standard MIMIC-CXR[10] test set of 3,269 studies. To create the clinical information prompt, the "PROVIDERS:" field was set to "Unspecified", the "PROCEDURE:" field was set to "XR CHEST", and the "HISTORY:" field was set to "None". The "COMPARISON:" field was set to the time difference between the study of interest and the most recent prior study contained in the MIMIC-CXR dataset for that patient based on the DICOM metadata. Model inference was otherwise performed using the hyperparameters previously described. Recently described models were identified via review of pre-prints and published literature for comparison. Further comparisons to unpublished models may be made using the ReXrank leaderboard (https://github.com/rajpurkarlab/ReXrank). In addition, because the entire MIMIC-CXR training dataset of 222,758 studies was used in development of all other models referenced here, model performance was benchmarked after finetuning the model on the MIMIC-CXR training set for 10 epochs using the hyperparameters and training strategy previously described, albeit with a lower learning rate of 1E-4, to provide a direct comparison.

© 2025 Huang J et al. *JAMA Network Open*.

All 95% confidence intervals for evaluations on the MIMIC-CXR test set were calculating using 500 bootstrap samples as previously described. [9]

Note that CheXbert, [8] RadGraph, [11] and RadCliQ were developed specifically for chest radiograph report evaluation, [9] while similar clinically-grounded metrics have not yet been proposed for evaluation of non-chest radiograph reports. However, these metrics are presented as a benchmark for future comparison, based on the ability of CheXbert and RadGraph to extract clinical entities relevant to non-chest radiographs in the internal test set.

© 2025 Huang J et al. *JAMA Network Open*.

eMethods 5: Ablation and model scaling evaluation

Ablation studies and the model scaling evaluation were performed by training on the MIMIC-CXR dataset, to ensure reproducibility. The standard train, validation, and test splits were used. Tiling and prompting were performed as previously described. A baseline model was trained using the same hyperparameters as used for original model training; the clinical information prompt was constructed using MIMIC-CXR data as described above. Ablation of tiling was performed by randomly selecting only one current and one prior image (if applicable) among the anteroposterior and posteroanterior views, if more than one image was present in a study; thus, the extent of image resizing remained consistent across the entire dataset. Ablation of prompting was performed by using a single prompt for each study, containing “Unspecified” for the provider, procedure, history, and comparison fields. Inference-time ablations of tiling and prompting were also performed by applying these ablations to the trained baseline model to perform inference.

Model scaling was investigated by separately training the model with a higher resolution vision encoder (ViT-base with patch size 8), as well as with a larger text decoder (OPT-350M).[4] Because the original model used a ViT-base checkpoint pre-trained using DINO,[3] for which only ViT-small and ViT-base checkpoints are available, scaling of the vision encoder was investigated by decreasing the patch size rather than directly increasing the encoder size (e.g., to ViT-large).

To assess potential variation in model performance due to the degree of image tiling present in the radiograph studies, information on number of image counts was retrieved from the EHR for the chest and non-chest prospective non-model usage test sets. Multiple regression models were fit to determine whether RadCliQ-v0 scores were associated with primary study and comparison study view counts for chest and non-chest studies.

© 2025 Huang J et al. *JAMA Network Open*.

eMethods 6: Model implementation and usage

To perform inference, relevant clinical data are automatically retrieved from EHR data; information not available to create the clinical data prompt is denoted as “Unspecified”. Upon receipt of an HL7 message associated with completion of radiograph acquisition, a server downloads all available DICOM format images for the imaging study, pre-processes them, and provides them as model input along with relevant clinical data from the HL7 message. An appropriate comparison study is automatically identified by querying the EHR for the most recent radiograph study examining the same body part. Inference is performed for all studies across the health system using a machine with one 80GB NVIDIA A100 GPU at a cost of approximately $4.00 per hour. Typical decoding[12] with a parameter value of 0.9 is used. To ensure uniform formatting of report outputs, chest and non-chest studies each use one standard radiologist name in the clinical information prompt. The model outputs are then populated within PowerScribe as custom fields ("metadata") and are included within a template selectable by radiologists, either manually or by voice command, who are using the model. Because inference completes within seconds, in the overwhelming majority of cases the model-generated report was available immediately upon opening a study. Documentation start time was defined to be the time at which the radiologist first opened the study.

All radiologists documented using voice dictation and individually created report templates within PowerScribe both before and during the model implementation period. Use of the tool is limited to attending radiologists, and radiologists must attest to independently interpreting the imaging and amending the draft report as needed, mimicking their workflow for revising trainee-produced reports. Importantly, draft AI reports are only visible to approved radiologists, unlike preliminary resident reads which are accessible to all providers, and at no point does the draft AI report serve as the sole interpretation for any imaging.

© 2025 Huang J et al. *JAMA Network Open*.

eMethods 7: Power analysis for peer review study

Considering the large sample size of reports included for documentation timing efficiency, it was not feasible to perform peer review for the entire dataset. Therefore, for the peer review study, an initial power analysis was conducted (G*Power v3.1.9.6, www.psychologie.hhu.de) to detect an effect size of 0.15 (similar in magnitude to a previous study[13]) with 80% power with fixed effects of model usage (model, no model) and study type (chest, non-chest). It was determined that a total sample size of 351 would be needed to detect the study type by model usage interaction effects to a small (0.3) effect size. A sample size of 400 randomly sampled studies was collected to allow for a 15% attrition rate due to radiologists being excluded from evaluating their own reads.

© 2025 Huang J et al. *JAMA Network Open*.

eMethods 8: Peer review evaluation scale and platform

The rating scales for peer review measurement of clinical accuracy and textual quality of radiograph reports were based on a previously published scale[13] but with separation of the clinical and textual quality components. A similar system is used for peer review of studies at our institution. Similarly to the RADPEER scoring system widely used in quality assurance for radiologist interpretations, the scale differentiates between reporting discrepancies with and without clinical significance. [14] In doing so, the scale separates studies by acceptability for informing clinical patient care.

To evaluate clinical accuracy and textual quality, radiologists were instructed to independently interpret the study of interest given the imaging data provided using their normal workflow, then evaluate clinical accuracy and text quality of the given report using the rating scales shown in Box 1. Instructions provided to the reviewers are shown in Box 2. Radiologists used a web application (eFigure 1) to perform all grading. Upon opening of a study, the relevant imaging and comparison studies automatically open in a test instance of Visage (Richmond, Australia). For each graded study, all radiographs of the same body part obtained up to a month prior were included, as well as related anatomy (e.g. for a wrist study, hand and forearm radiographs were included, as well as contralateral studies). Additionally, the five most recent radiographs of the same body part were identified and included, if not already contained within the one-month interval. Moreover, if the graded study specified a comparison study that was not already identified by the prior steps, then that study was separately identified and included. All report text, image, and Digital Imaging and Communications in Medicine (DICOM) data was deidentified, with random shift of dates (eFigure 1) and redaction of any other identifying information. The same date shift was used for all comparison studies so that relative time intervals remained accurate. If a clinical score of less than four was given, additional prompts appeared for the radiologist to specify all reasons for disagreement.

© 2025 Huang J et al. *JAMA Network Open*.

Box 1: Instructions provided to radiologists performing peer-review evaluation of imaging studies.

A. Clinical accuracy ratings
    a. For clinical accuracy, consider how the clinical findings pertinent to the case are presented by the report, based on your independent interpretation of the imaging study
        i. A score of 4 indicates complete agreement with the clinical findings contained in the study
        ii. A score of 3 indicates that critical findings are appropriately reported, but one or more non-critical findings are not
        iii. A score of 2 indicates that non-critical findings are appropriately reported, but one or more critical findings are not
        iv. A score of 1 indicates that both critical and non-critical findings are inappropriately reported, or that the majority of study findings are inappropriately reported
    b. A critical finding is defined to be any finding in the imaging study which would change the immediate management of the patient if reported incorrectly, in your clinical judgement
    c. If the clinical accuracy score is <4, please indicate all reasons for your disagreement using the checkboxes, and provide a brief explanation for each discrepancy (e.g. "Pneumothorax is on the right, not left. A left pleural effusion is not reported.")
B. Text quality ratings
    a. For textual quality, only consider aspects of the report text itself (i.e., imagine that there are no images available, and you are only judging the quality of the report text in isolation)
        i. A score of 3 indicates that no changes to grammar, wording, or formatting are needed
        ii. A score of 2 indicates that the report requires changes which could reasonably be revised in the typical clinical workflow
        iii. A score of 1 indicates that the report requires extensive changes which necessitate rewriting the entire report from scratch

© 2025 Huang J et al. *JAMA Network Open*.

eMethods 9: Pneumothorax prioritization strategy

The prioritization system was designed to identify radiograph studies for which the AI draft and available EHR indicated a clinically significant, new pneumothorax. This ran in a “shadow deployment” in real time alongside the live model deployment workflow. Thus, studies were prioritized in real time, but no clinician notification occurred; rather, information on flagged studies was logged to the AI model monitoring database. All studies for which a model-generated report was available underwent prioritization. This included both chest and non-chest studies due to the possibility of a pneumothorax being visualized on a shoulder, rib, or abdominal radiograph.

The prioritization strategy is as follows. First, reports which did not contain the case-insensitive substring “pneumothora” were excluded. Next, studies with a reason for exam or for which the comparison study reason for exam contained any “Reason for exam” substring given in eTable 1 were excluded. Moreover, studies were excluded if the patient had been admitted to any surgical unit or the cardiothoracic intensive care unit within the past four days. At this point, the model-generated report text was classified as containing or not containing a pneumothorax using RadGraph[11] to identify presence of any of the following clinical entities: “pneumothorax”, “pneumothoraces”, “hydropneumothorax”, “hydropneumothoraces”. Both “uncertain” and “definitely present” entities, per RadGraph, were considered to indicate presence of a pneumothorax; studies not containing a pneumothorax per RadGraph were excluded. Next, patients with known pneumothoraces were excluded by examining the model and any prior imaging report text in the past four days for any “Chest tube” substring given in eTable 1, and were also excluded if a pneumothorax was present, per RadGraph. Finally, to avoid prioritizing clinically insignificant or stable pneumothoraces, studies were excluded if the model report contained any sentence with both the substring “pneumothora” as well as one of the “Clinical context” substrings.

To identify the ground truth set of studies with clinically significant pneumothorax for which a radiologist notified the clinical care team, all studies interpreted during the live prioritization period of February 5, 2024 to April 24, 2024 were retrospectively examined. Reports were searched for instances of clinical team notification by performing a text search for a custom tag which radiologists at our institution use to indicate presence of a critical finding, or any of the following case-insensitive substrings: “discussed”, “sent”, “text”, “called”, “page”, “notif”, “communic”, “given to”, “relay”, “convey”, “delivered”, “ aware”, “talk”. The resultant reports were parsed using RadGraph to identify those containing a pneumothorax, as above, and prioritized using the system previously described. Flagged studies for which report text contained both a pneumothorax and documentation of clinical team notification were confirmed by manual review. Time to clinical team notification was calculated as the interval from study acquisition completion to documented time of this notification, if provided in the report text; if not, the time at which the study was first opened by the radiologist was used, as logged in electronic health record data.

© 2025 Huang J et al. *JAMA Network Open*.

eMethods 10: Secondary statistical analyses

To further examine the relationship between potential factors influencing documentation time with the AI model, a multiple regression model was fit (*stats package in R, v4.3.0*) to documentation time with potential predictor values of: number of word edits, RadGraph F1[9] scores (to measure extent of clinically relevant content edited), and study type with covariates of RadGraph[11] entity and relation count (to measure clinical content of the report), the original AI-generated report word count, study critical or non-critical status, radiologist, proportion of radiologist pre-model reads with a resident, and years of radiologist clinical practice experience. All non-significant covariates were removed from the model. Word count was calculated by first extracting the Findings and/or Impressions sections of the report text (excluding header study background information and footer attestation or signature text). Word edits were calculated by extracting the Findings and/or Impressions sections, then using the *difflib* module (Python version 3.10.6) to count word-level differences between the AI model and final radiologist reports. Word edit count was used instead of word error rate as a measure of report editing as the latter resulted in a lower model R^2 value and greater residual sum of squares; moreover, word error rate may be derived from word edit count and generated report length, both of which were included in the model. RadGraph information quantity was defined as the total number of anatomy and relation entities identified in the model-generated report within the clinical entity graph produced by RadGraph. RadGraph F1 was calculated using a standard library[9] by comparing model reports to final radiologist reports as the ground truth. "Critical" studies were defined to be those containing any of the following CheXbert pathology categories: "Enlarged cardiomediastinum", "Lung opacity", "Consolidation", "Pneumonia", "Pneumothorax", and "Fracture", while "Non-critical" studies were all other studies; this categorization was arrived at by radiologist consensus.

The distributions of radiograph type were compared between pre- and post-model datasets using a Chi-Square Goodness of Fit Test after categorizing all radiographs into the following categories: chest, abdomen/pelvis, spine, lower extremity, upper extremity, thorax-musculoskeletal, and other. Because a single radiograph could appear in multiple categories, Chi-Square tests were performed individually for each body part with Bonferroni-Holm corrections.

Subgroup analyses were also completed to evaluate potential differences in peer review quality scores by pathology with a cumulative link mixed model fit with the clinical score on reports subgrouped by presence of CheXbert pathology categories. Comparisons between subgroups are reported on the logit scale. The "Consolidation" category was not included in this subgroup analysis due to limited data points. To investigate the types of errors, a generalized logistic mixed-effects model was fit on the binary response variable with fixed effects of time and study type. Kendall's W was employed to calculate clinical score- and text score rating concordance using DescTools (version 0.99.49) in R using corrections for tied rankings.

© 2025 Huang J et al. *JAMA Network Open*.

eAppendix 1: Control group radiologist documentation efficiency

The control group of studies interpreted by the cohort of radiologists with no model usage consisted of a pre-model implementation and a post-model implementation set of studies, both of which comprised 10,897 studies of which 8,683 (79.7%) were chest and 2,214 (20.3%) were non-chest radiographs. The chest radiographs were interpreted by 12 radiologists reading a median of 204 studies (IQR 49.5-924) and the non-chest radiographs were interpreted by 15 radiologists reading a median of 60 studies (IQR 28-122).

In the non-model control cohort, there was a significant main effect of procedure type on documentation time (χ^2=14.58, df=1, *P*<0.001), with documentation time for non-chest studies being significantly slower than chest studies (by 133.0±34.8 s). The main effect of model usage was not significant (χ^2=3.65, df=1, *P*=0.06), with studies before (184±25.0 s) not being significantly different than studies after model implementation (194.0±25.0 s). The time by study type interaction was not significant (χ^2=0.26, df=1, *P*=0.61). Thus, there was no evidence for any change in documentation efficiency during the study period for radiologists who did not use the model.

© 2025 Huang J et al. *JAMA Network Open*.

eAppendix 2: Factors associated with documentation efficiency improvement

We hypothesized that documentation efficiency might be associated with measures of generated report content (RadGraph information quantity and word count) and quality (RadGraph F1 score and word edit count), due to time required to verify report; with presence of critical findings in reports, due to study complexity; and with radiologist seniority and experience editing trainee reports, due to expertise and familiarity with editing in the clinical workflow. On secondary analysis investigating the association of these factors with study documentation time, the multiple regression model was significant (F=145.0, P<0.001, adjusted R^2=0.25). Word edit count was significantly associated with documentation time (1.16±0.07, t=17.1, P<0.001) although RadGraph F1 was not. RadGraph information quantity (1.50±0.08, t=19.0, P<0.001), model-generated report word count (-0.56±0.07, t=-8.09, P<0.001), and non-chest procedure type (51.6±8.95, t=5.76, P<0.001) were significantly associated with documentation time, while critical finding presence, years of experience, and proportion of pre-model reads interpreted with a resident were not.

© 2025 Huang J et al. *JAMA Network Open*.

eAppendix 3: Peer review radiologist information

Among the 800 reviewed studies, 10 radiologists were represented (6 reading chest, 1 reading non-chest, and 3 reading both study types). The chest studies were evaluated by two generalist and two cardiothoracic subspecialty-trained radiologists, and the non-chest studies by four musculoskeletal subspecialty-trained radiologists. Raters had a median of 6.5 years of post-residency practice experience (range: 3-17 years).

© 2025 Huang J et al. *JAMA Network Open*.

eAppendix 4: Cumulative link mixed model outputs for textual quality peer review

The main effect of study type was significant (χ^2=11.59, df=1, *P*=0.001), with chest studies rated significantly higher than non-chest studies (by 1.37±0.82, z=3.25, *P*=0.001). The model usage by study type interaction was also significant (χ^2=5.05, df=1, *P*=0.02). Post-hoc tests revealed that chest textual ratings were significantly greater with the model compared to without (by 0.83±0.57, z=2.9, *P*=0.02). Chest studies with the model were also rated significantly greater than non-chest studies with (by 1.77±0.92, z=3.7, *P*=0.001) and without (by 1.80±0.92, z=3.8, *P*=0.001) the model. No other comparisons were significantly different.

© 2025 Huang J et al. *JAMA Network Open*.

eAppendix 5: Evaluation by automated metrics of radiograph quality

Accuracy of the model-generated reports for pathology classification for both chest and non-chest studies was investigated by using the CheXbert labeler (eFigure 2), demonstrating performance competitive with or exceeding that of recently reported models across individual pathologies and aggregated macro-F1 score. [15-19] Overall model performance for report generation was characterized by evaluation on the internal held-out and prospective test sets as well as the external MIMIC-CXR test set (eTable 7), showing competitive performance of our model compared to recently reported approaches. [16-21]

Moreover, ablation studies were performed to characterize the impact of model architecture design on performance, as measured by automated metrics of report quality (eFigure 4). Ablation studies were performed by training the model from scratch on the MIMIC-CXR dataset, to enable independent replication. Overall model performance was decreased substantially with ablation of prompting but remained consistent with ablation of tiling, though performance was higher with tiling than without for studies containing multiple views. Similarly, ablation of prompting during inference decreased overall performance while ablation of tiling during inference did not.

Additionally, considering the relatively small size of the model compared to recently reported approaches to radiograph report generation, we investigated the effect of increasing the model size on performance, using the MIMIC-CXR dataset (eFigure 4). These experiments show that increasing encoder resolution and decoder size both improve model performance.

eFigure 1: Peer review web application

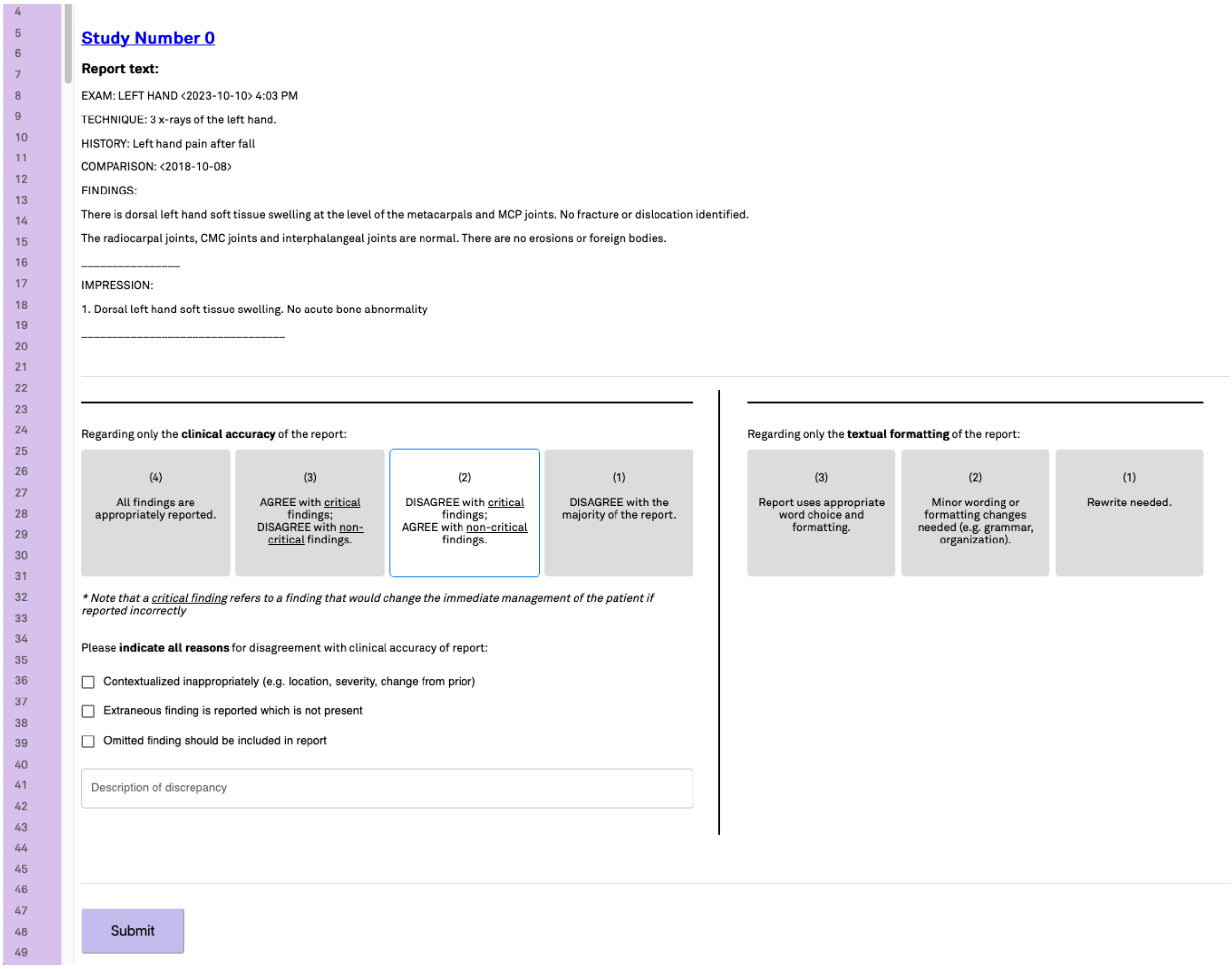

Screenshot of the peer review web application used by radiologists to grade report quality. The checkboxes and text entry field for description of discrepancy are only shown when a rating of less than four is selected for clinical accuracy. Note that all dates were shifted by a randomized interval.

© 2025 Huang J et al. *JAMA Network Open*.

eFigure 2: Model performance across pathology subgroups

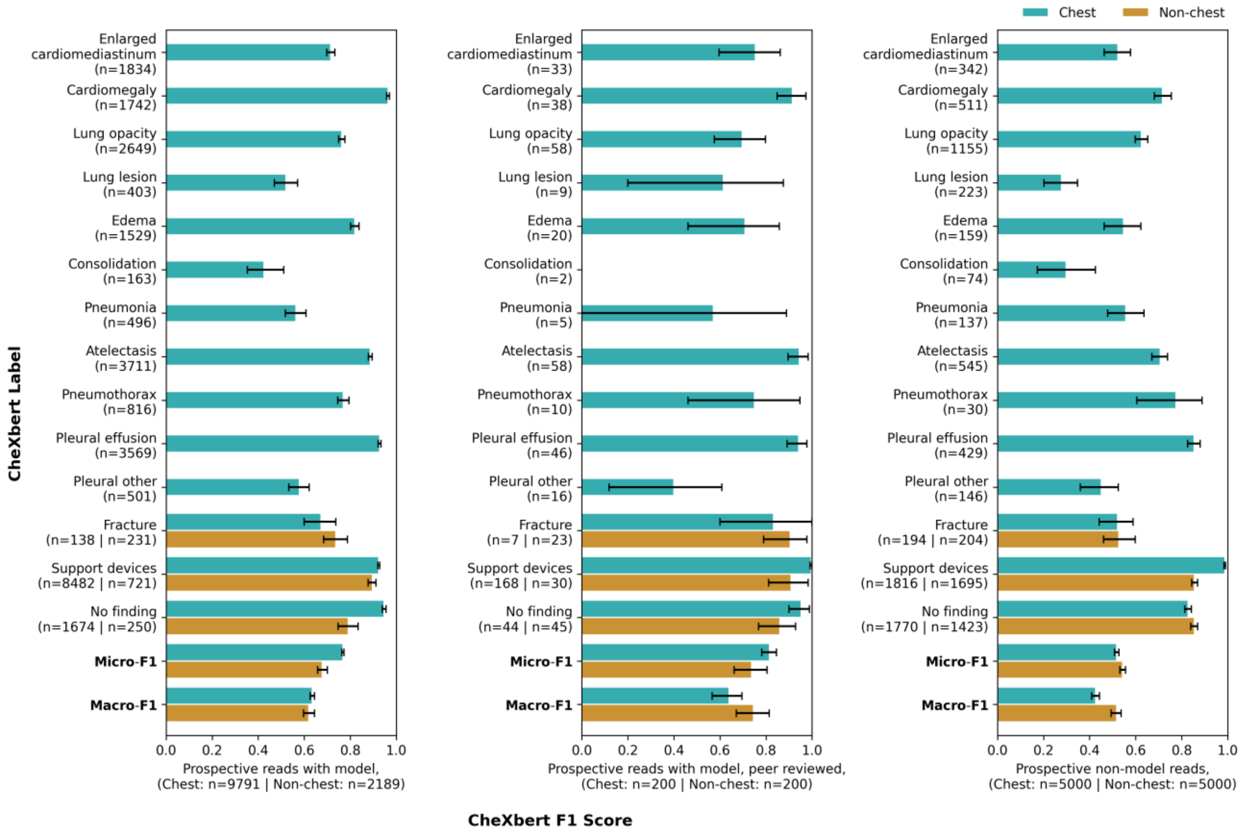

Model performance was evaluated across pathology subgroups by calculating CheXbert F1 scores as well as micro- and macro-averaged F1 scores based on outputs of the CheXbert labeler, for the prospective model usage dataset (left), the prospective peer review quality evaluation subset (center), and the prospective test dataset of reads documented without model usage (right). F1 scores were calculated using model-generated reports as the prediction and final radiologist reports, either derived by editing the model-generated report (left and center panels) or independently documented without knowledge of the model-generated reports (right), as ground truth. The prospective non-model macro-F1 score of 0.426 for chest studies, which measures overall performance averaged across all 14 pathology categories, demonstrates performance competitive with or exceeding the previously reported state of the art. [15-17,21] For subgroups including both chest and non-chest studies, the chest study count is listed first, followed by the non-chest study count.

© 2025 Huang J et al. *JAMA Network Open*.

eFigure 3: Documentation time change by radiograph subgroup

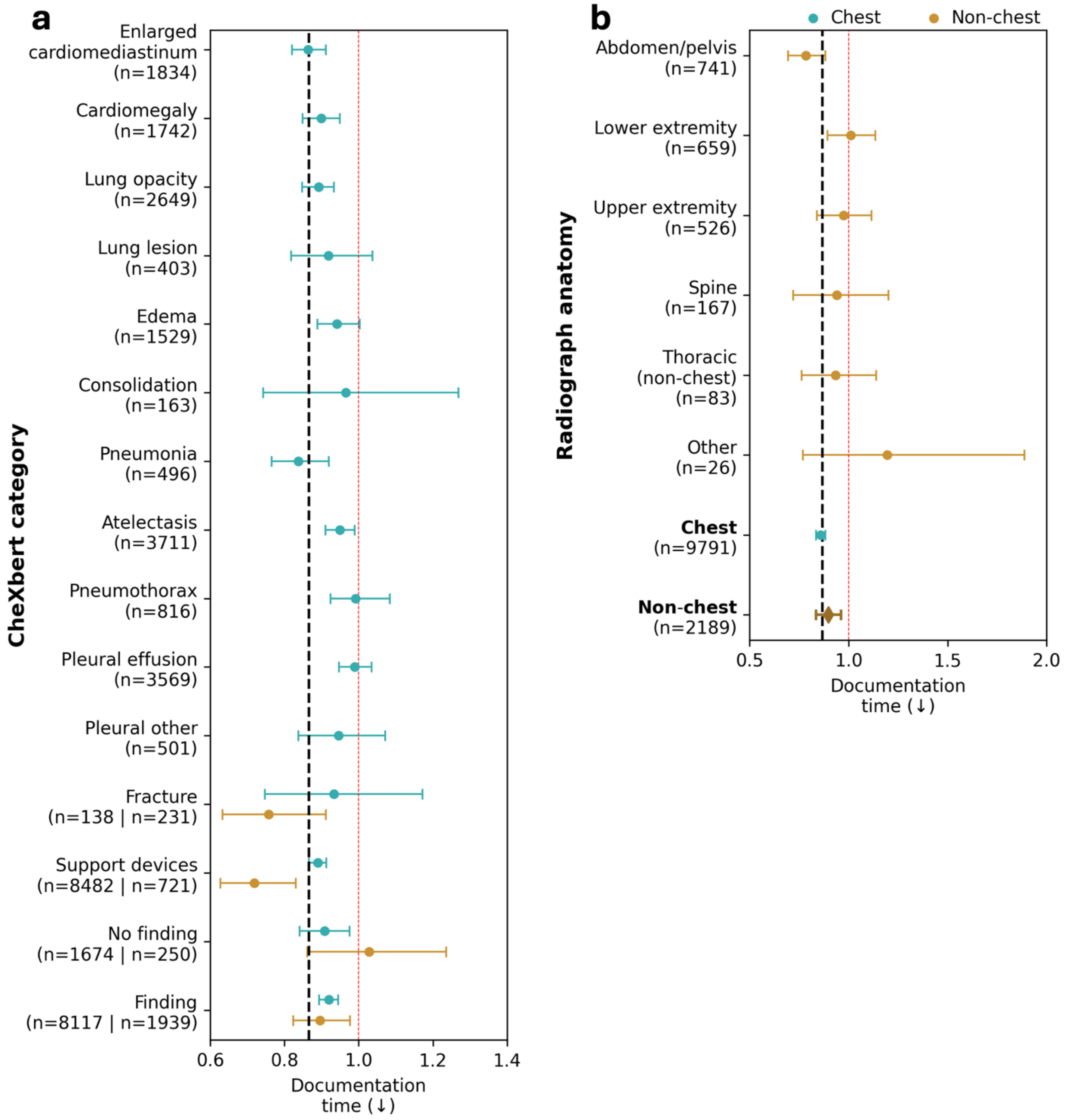

Documentation time improvement as ratio of post-model documentation time to pre-model documentation time by pathology subgroups (left) and anatomy subgroups (right) of the prospective timing dataset (n=11,980 studies) identified using the CheXbert labeler. Data are presented as means and 95% confidence intervals were computed using 500 bootstrap samples. Only the “Fracture”, “Support devices”, “No findings”, and “Finding” categories are considered for non-chest studies, as the others are not relevant to non-chest studies. The dashed black line shows the overall documentation time over the entire dataset. The dotted red line indicates no

© 2025 Huang J et al. *JAMA Network Open*.

change in documentation time, with data points to the left reflecting faster documentation with the AI model (indicated by the downward arrow, ↓). For subgroups including both chest and non-chest studies, the chest study count is listed first, followed by the non-chest study count. Diamond markers indicate that the data point is an aggregate of other subgroups shown above. A comprehensive breakdown of radiograph anatomy types is provided in eTable 4.

© 2025 Huang J et al. *JAMA Network Open*.

eFigure 4: Pneumothorax flag criteria

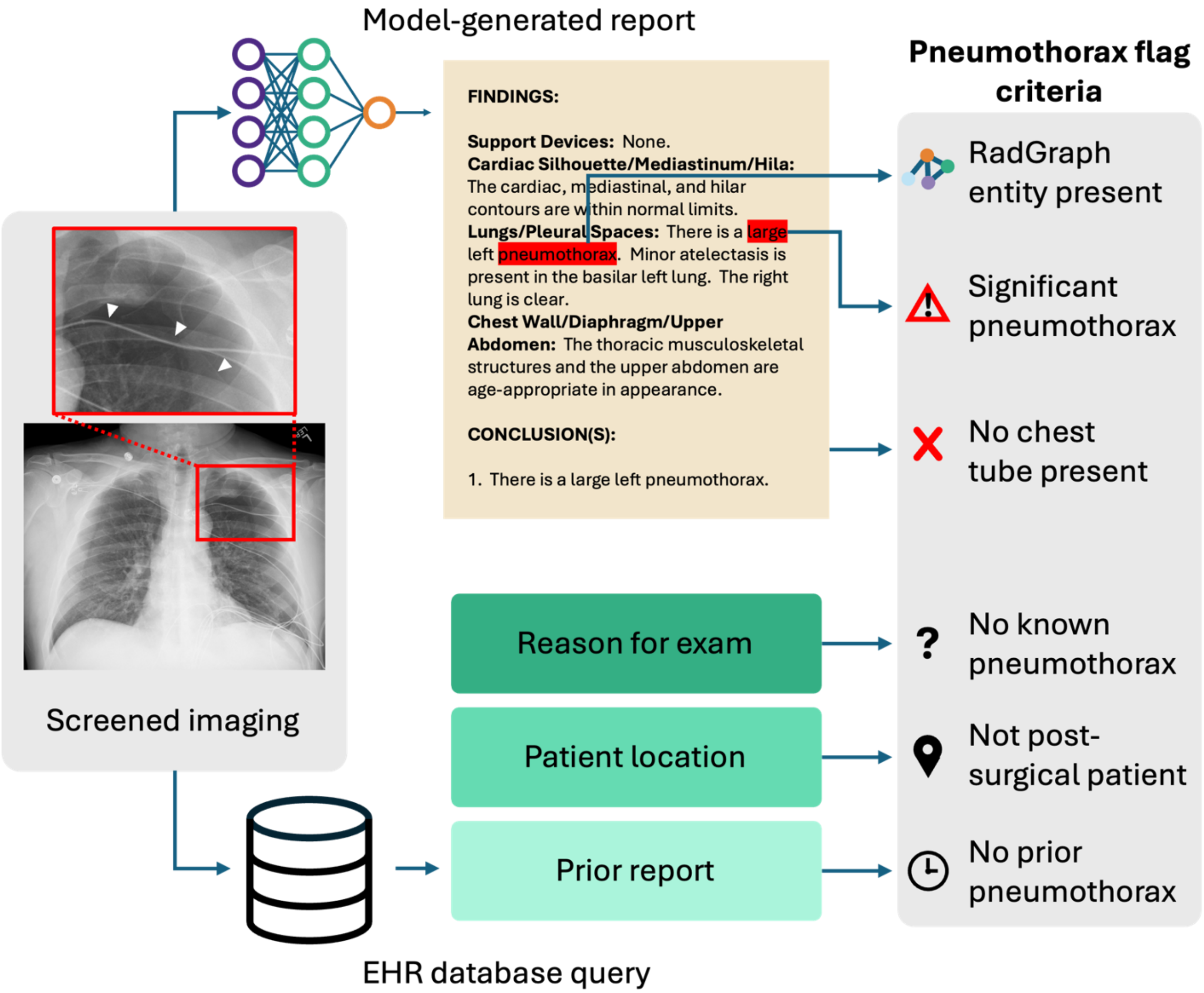

Radiograph studies containing a clinically significant and unexpected pneumothorax (red inset) are identified in real time by examination of the AI model-generated report and relevant data from the hospital EHR. The screening criteria were designed to maximize relevance of prioritized pneumothoraces by excluding patients with known pneumothoraces, patients who recently underwent surgery, and patients with subtle or radiographically uncertain pneumothoraces unlikely to impact clinical care.

© 2025 Huang J et al. *JAMA Network Open*.

eFigure 5: Ablation studies and model scaling investigation

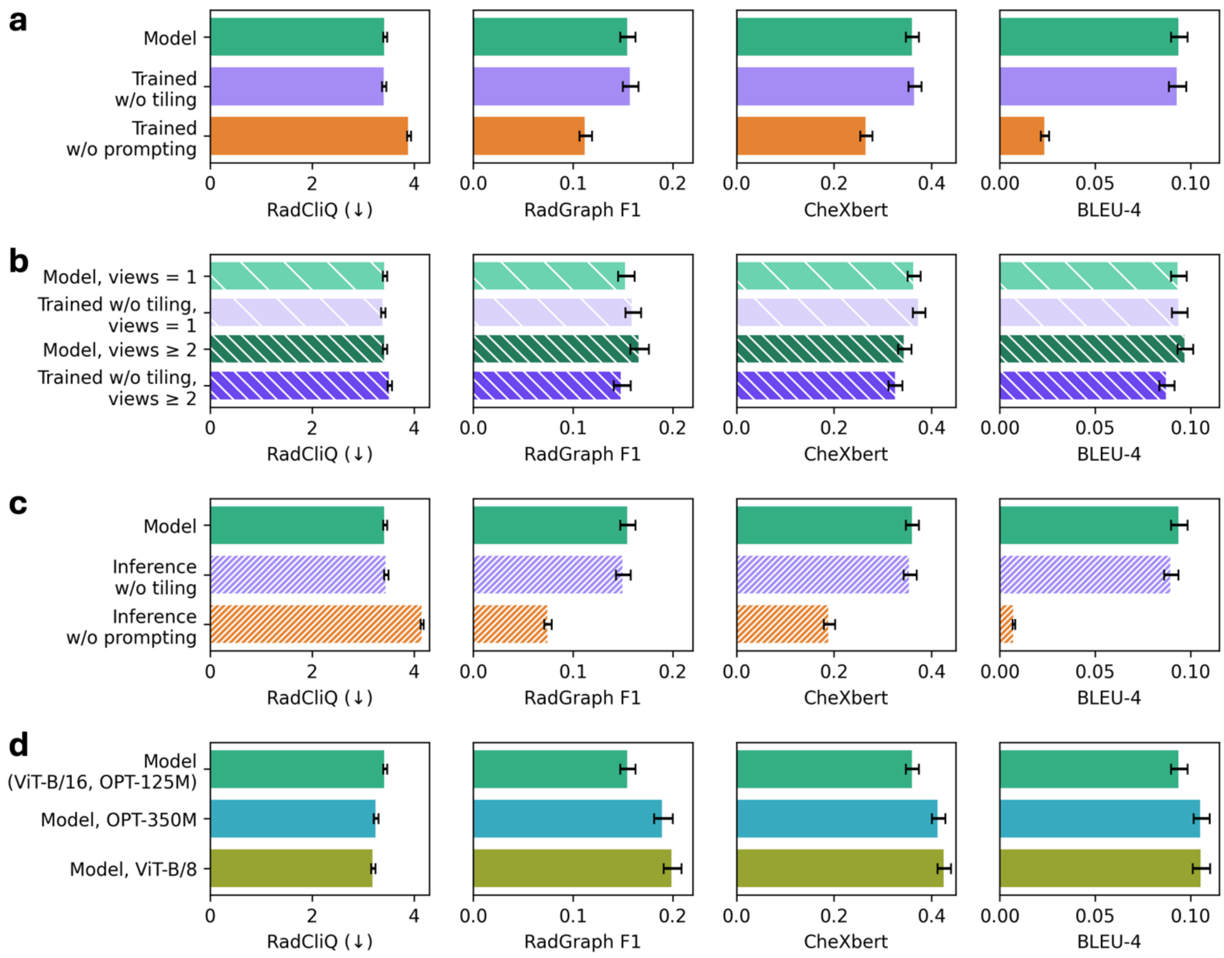

a, Impacts of image tiling and custom prompting were investigated via ablation studies performed by training the model from scratch on the MIMIC-CXR database. When trained without tiling, the model demonstrates comparable performance to the baseline across all four evaluated metrics. However, the model trained without prompting demonstrates degraded performance. **b**, Subgroups of the test set based on number of views in the current study views demonstrate slightly improved performance for studies with multiple views when the model is trained with tiling, compared to without. **c**, Inference-time ablations of tiling and prompting demonstrate similar performance without tiling but worse performance without prompting. **d**, Scaling model size by increasing encoder resolution or increasing decoder size improves performance.

© 2025 Huang J et al. *JAMA Network Open*.

eFigure 6: Flowchart for pneumothorax-flagging study inclusion

Shown is the cohort of model-generated radiograph reports screened for presence of clinically significant and unexpected pneumothorax, along with numbers of inclusions and exclusions by flag criteria.

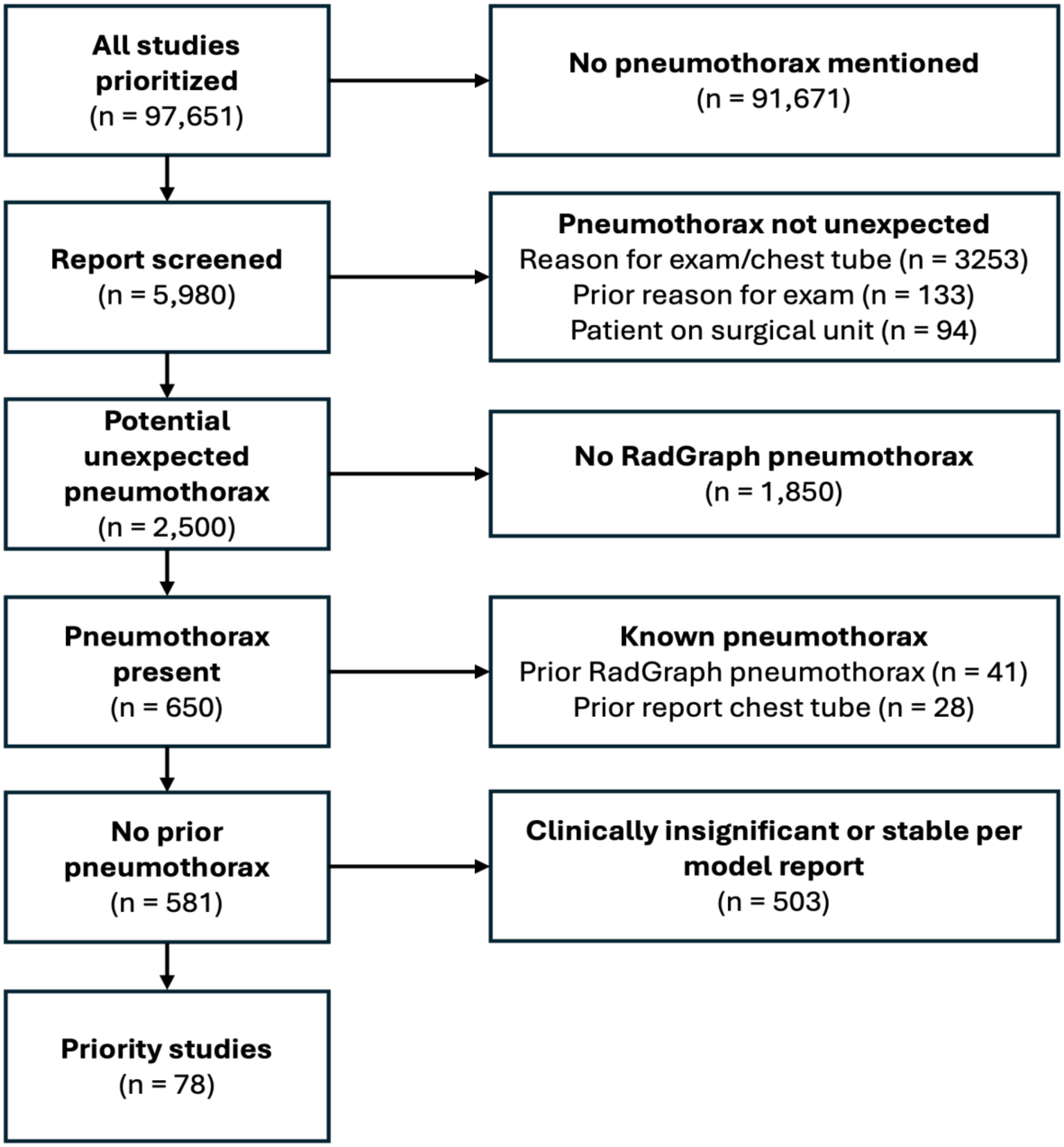

© 2025 Huang J et al. *JAMA Network Open*.

eTable 1: Prioritization text search exclusions

Clinical information	Substring
Chest tube	“chest tube”, “pigtail”, “pleural catheter”, “pleurx”
Reason for exam	“chest tube”, “transplant”, “thoracic decompress”, “thoracent”, “postop”, “post op”, “post-op”, “ surg”, “postsurg”, “ oht”, “ ct remov”, “lung tx”, “biopsy”, “ bx”, “lobectom”, “ vats”, “wedge resec”, “segmentectomy”, “pleurodesis”, “follow-up pneumothorax”, “follow up pneumothorax”, “fu pneumothorax”, “f/u pneumothorax”
Clinical context	“decrease”, “stable”, “smaller”, “improve”, “resolv”, “resolution”, “suggestion”, “subtle”, “minimal”, “tiny”, “trace”

Substring search (case insensitive) used to exclude model-generated report text and clinical information.

© 2025 Huang J et al. *JAMA Network Open*.

eTable 2: Study demographic information

	Study cohort		
	Documentation efficiency (n=23,960)	Quality peer review (n=800)	Pneumothorax flagging (n=97,651)
**Number of unique patients**	14,460	800	73,881
**Age, mean (SD), y**	59.6 (17.5)	57.5 (19.6)	60.5 (18.1)
**Sex, No. (%)**			
Female	11,689 (48.8)	457 (57.1)	54,088 (55.4)
Male	12,268 (51.2)	343 (42.9)	43,535 (44.6)
Other	3 (<0.1)	0	19 (<0.1)
Unknown	0	0	9 (<0.1)
**Race and ethnicity, No. (%)**			
American Indian or Alaska Native	60 (0.3)	3 (0.4)	344 (0.4)
Asian	995 (4.2)	28 (3.5)	3,592 (3.7)
Black	4,498 (18.8)	127 (15.9)	12,135 (12.4)
Hispanic	4,408 (18.4)	145 (18.1)	5,020 (5.1)
Native Hawaiian or Other Pacific Islander	38 (0.2)	1 (0.1)	233 (0.2)
White	12,822 (53.5)	462 (57.8)	62,979 (64.5)
None of the above	896 (3.7)	25 (3.1)	8,365 (8.6)
Unknown	243 (1.0)	9 (1.1)	4,983 (5.1)
**Clinical setting, No. (%)**			
Emergency Department	3,015 (12.6)	177 (22.1)	26,184 (26.8)
Immediate Care	1,985 (8.3)	118 (14.8)	11,375 (11.6)
Intensive Care Unit	7,920 (33.1)	102 (12.8)	15,846 (16.2)
Other Inpatient	4,531 (18.9)	75 (9.4)	13,987 (14.3)
Other Outpatient	6,509 (27.2)	328 (41.0)	30,259 (31.0)
**X-ray machine manufacturer, No. (%)**			
Carestream	20,628 (86.1)	666 (83.3)	54,926 (56.2)
EOS	113 (0.5)	7 (0.9)	321 (0.3)
GE Healthcare	989 (4.1)	50 (6.3)	22,112 (22.6)
Kodak	1,717 (7.2)	36 (4.5)	1,737 (1.8)
Siemens	227 (0.9)	21 (2.6)	1,837 (1.9)
Philips	275 (1.1)	20 (2.5)	1,350 (1.4)
Other	11 (<0.1)	0	15,368 (15.7)

Demographic information for the documentation efficiency, quality peer review, and pneumothorax flagging cohorts of radiograph studies. The sampling strategies for each cohort are described in the manuscript and eMethods. “Other” manufacturers included: Agfa, Canon, DRGEM, Duet, Fujifilm, Hologic, Konica Minolta, Rayence, Thales, and Toshiba.

© 2025 Huang J et al. *JAMA Network Open*.

eTable 3: Training and evaluation set radiograph breakdown by anatomy.

	Anatomy	Training Set (n=4,186,288)	Held-out Test Set (n=10,000)	Prospective Internal Test Set (n=10,000)	Prospective Model-Assisted Reads (n= 11,980)	Matched Pre-Model Reads (n=11,980)	Pre-Model Quality Evaluation Reads (n=400)	Prospective Quality Evaluation Reads (n=400)
**Chest**	chest	2,127,117	5,000	5,000	9,791	9,801	200	200
**Lower extremity**	foot	178,886	437	462	178	186	21	27
	ankle	125,870	300	283	97	107	12	15
	knee	217,182	509	830	301	279	29	38
	tibia/fibula	40,460	111	80	34	30	3	7
	femur	24,667	51	47	33	26	1	4
	toe	17,460	47	37	16	15	0	1
**Abdomen/ Pelvis**	abdomen	267,438	668	415	447	574	10	3
	pelvis	202,287	514	592	297	247	24	24
**Upper extremity**	shoulder	134,107	307	486	226	157	21	23
	hand	122,632	284	253	108	133	30	17
	wrist	111,718	274	230	78	102	15	8
	finger	70,626	181	172	50	46	6	3
	elbow	57,763	123	94	46	47	7	8
	forearm	27,509	73	23	15	12	1	1
	humerus	17,377	40	37	18	15	2	4
**Spine**	spine	331,902	838	937	167	180	21	16
**Thoracic, non-chest**	ribs	47,451	101	163	81	53	6	15
	sternum	1,721	4	1	2	1	0	0
**Other (n=149,254)**	other/ unspecified	63,037	151	8	0	1	0	0
	intraoperative	27,631	66	12	6	8	0	1
	babygram	26,249	43	0	0	0	0	0
	neck	10,433	25	14	2	3	0	1
	hardware evaluation	9,914	23	9	10	11	0	1
	bone age	5,525	14	0	0	0	0	0
	metastasis survey	2,572	0	2	3	4	0	1
	facial	2,270	9	19	3	2	1	0
	jaw	1,623	5	6	2	1	0	1

Note that the anatomy subgroup counts sum to greater than the number of studies as some studies pertain to multiple body parts.

© 2025 Huang J et al. *JAMA Network Open*.

eTable 4: Timing data per radiologist

Radiologist ID	Read count	Mean read time prior to model (s)	Mean read time with model (s)	Read time speedup using model (s)	Read types
**1**	3267	131.2	128.4	2.83	chest*
**2**	2993	143.5	84.8	58.7	chest*
**3**	1927	139.7	138.9	0.84	chest*
**4**	1072	133.3	135.8	-2.43	chest*, non-chest*
**5**	608	85.2	77.5	7.77	chest*
**6**	501	100.6	122.0	-21.4	chest*, non-chest*
**7**	306	95.4	110.4	-15.0	chest*
**8**	301	112.2	82.0	30.2	non-chest*
**9**	183	135.9	152.8	-17.0	non-chest
**10**	126	421.3	301.3	120.0	non-chest
**11**	126	73.6	50.2	23.3	chest*, non-chest*
**12**	117	136.4	126.4	10.0	non-chest
**13**	98	262.8	272.2	-9.47	chest*
**14**	67	187.0	119.4	67.6	non-chest
**15**	60	234.2	157.9	76.3	non-chest
**16**	60	346.6	182.3	164.3	non-chest
**17**	34	160.9	126.7	34.1	chest, non-chest
**18**	30	147.0	128.8	18.3	non-chest
**19**	29	898.3	706.5	191.8	non-chest
**20**	29	69.4	66.7	2.76	chest, non-chest
**21**	26	124.9	187.8	-62.9	non-chest
**22**	20	66.5	119.9	-53.4	chest

Read types indicated with an asterisk were included in the peer review study, as the radiologist had accrued at least 10 model-assisted reads of that type by March 14, 2024.

© 2025 Huang J et al. *JAMA Network Open*.

eTable 5: Example model-generated reports and radiologist edits

Procedure type	Reason for exam	Edited model report
Chest AP Portable	Post chest tube removal	CONCLUSION(S)<br><br>Support Devices: The small diameter right basilar chest tube has been removed. The tracheostomy tube, the NG tube, the right IJ central line, and the right PICC remain in place. The orphaned electrode fragment in the left subclavian vein ~~distribution and~~ is stable.<br>Cardiac Silhouette/Mediastinum/Hila: Cardiomegaly is stable. Calcific thoracic aortic atherosclerosis is present.<br>Lungs/Pleural Spaces: Right pneumothorax is smaller than on the prior study. Minimal left pleural effusion is stable. Interstitial pulmonary edema ~~has improved.~~ is unchanged. Subsegmental atelectasis is present at the lung bases.<br>Chest Wall/Diaphragm/Upper Abdomen: The thoracic musculoskeletal structures and the upper abdomen are unchanged.
Chest AP Portable	Status post cardiac surgery	CONCLUSION(S)<br><br>Support Devices: The endotracheal tube, the NG tube, the right IJ Swan-Ganz catheter, the 3 mediastinal drains, and the temporary epicardial pacemaker electrodes remain in place.<br>Cardiac Silhouette/Mediastinum/Hila: The postoperative cardiac silhouette is upper normal in size. A mitral valve bioprosthesis is present. Postoperative pneumomediastinum is present.<br>Lungs/Pleural Spaces: Small bilateral hydropneumothoraces are present. ~~present, the apical gas component of which is new.~~ There is atelectasis in both lower lobes.<br>Chest Wall/Diaphragm/Upper Abdomen: The thoracic musculoskeletal structures and the upper abdomen are unchanged.
Chest AP Portable	Cardiac evaluation	FINDINGS<br><br>Support Devices: None.<br>Cardiac Silhouette/Mediastinum/Hila: The cardiac, mediastinal, and hilar contours are within normal limits.<br>Lungs/Pleural Spaces: There is bibasilar subsegmental atelectasis. No effusion or pneumothorax is seen.<br>Chest Wall/Diaphragm/Upper Abdomen: There is pneumoperitoneum, likely postoperative. There is left chest wall subcutaneous emphysema.<br><br>CONCLUSION(S)<br><br>1. Pneumoperitoneum, likely postoperative.<br>2. Bibasilar subsegmental atelectasis.
Chest PA Lateral	Assess for pneumonia	FINDINGS<br><br>Support Devices: Aortic stent graft and surgical clips at the left neck. EKG leads overlie the patient.<br>Cardiac Silhouette/Mediastinum/Hila: Stable cardiomediastinal contour. ~~Stable cardiomediastinal contour.~~ Aortic silhouette prominent/aneurysmal as on prior CT <DATE>. Radiographics appearance of aortic stent similar to previous.<br>Lungs/Pleural Spaces: Linear opacities at the lateral left lung base. No new dense focal consolidation. No pleural effusion or pneumothorax.<br>Chest Wall/Diaphragm/Upper Abdomen: The thoracic musculoskeletal structures and the upper abdomen are stable including elevated left hemidiaphragm. Upper abdomen is unremarkable.<br><br>CONCLUSION(S)<br><br>1. Left basilar opacity may represent atelectasis or scarring. No new dense focal consolidation.

© 2025 Huang J et al. *JAMA Network Open*.

Procedure type	Reason for exam	Edited model report
Chest PA Lateral	Lung transplant candidate	CONCLUSION(S)<br><br>Support Devices: None.<br>Cardiac Silhouette/Mediastinum/Hila: Cardiac size is normal. Central pulmonary artery dilatation is present. There is calcific thoracic aortic atherosclerosis.<br>Lungs/Pleural Spaces: The lungs are hyperinflated. Ill-defined opacities at the right lung base could represent atelectasis but pneumonia or aspiration not excluded. There are reticular opacities of the lung bases suggestive of fibrosis. ~~is a poorly defined nodular focus of opacity in the lateral periphery right middle lobe that is new from the prior study. Subtle peripheral reticular opacity is present in the periphery of the lungs.~~ Pleural spaces clear. No pneumothorax. ~~There is blunting of the right costophrenic angle.~~<br>Chest Wall/Diaphragm/Upper Abdomen: The bones are demineralized.
Left femur, 2 views	Fall	FINDINGS<br><br>No acute fracture or dislocation is identified. There is a left total knee arthroplasty without evidence of hardware failure. There is incompletely assessed ~~lower lumbar spine posterior fusion. There is incompletely assessed~~ left hip osteoarthritis. There are vascular calcifications.<br><br>CONCLUSION(S)<br><br>No acute osseous finding.
Left 3rd finger, 3 views	Finger injury	There is dorsal dislocation of the third middle phalanx at the proximal interphalangeal joint. There is adjacent soft tissue swelling.
Left shoulder, 2 views	Acute pain of left shoulder	FINDINGS<br><br>~~There is~~ Postsurgical changes of a total left shoulder ~~hemiarthroplasty.~~ arthroplasty. The prosthetic humeral head is in expected position and alignment. There is no evidence of periprosthetic fracture. There is cortical irregularity along the inferior aspect of the glenoid, likely secondary to ~~heterotopic ossification.~~ remodeling and bone spur. There is no significant joint effusion.<br><br>CONCLUSION(S)<br><br>Status post left shoulder hemiarthroplasty.
Right knee, AP Lateral	New right knee pain, limited range of motion	FINDINGS<br><br>There is no acute fracture or osseous malalignment. ~~The femorotibial and patellofemoral~~ Mild tricompartmental osteoarthritis of the right knee. Mild joint ~~spaces~~ space ~~are preserved.~~ narrowing of the medial tibiofemoral compartment. There is small osteophytes formation in the patella. There is a small suprapatellar joint effusion. There is diffuse vascular calcification.<br><br>CONCLUSION(S)<br><br>1. No acute osseous finding.<br>2. Small joint effusion.<br>3. Mild ~~patellofemoral~~ tricompartmental ~~osteoarthritis.~~ osteoarthritis of the right knee.
Abdomen AP	Nausea	FINDINGS<br><br>LINES OR TUBES: None<br>LUNG BASES: The lung bases are clear.<br>BOWEL GAS PATTERN: There are no abnormally dilated loops of bowel. There is a moderate colonic stool burden.<br>CALCIFICATIONS/OTHER: An intrauterine device projects over the pelvis.

© 2025 Huang J et al. *JAMA Network Open*.

Procedure type	Reason for exam	Edited model report
		MUSCULOSKELETAL: There is posterior spinal fusion hardware at the L1-L2 level.<br><br>CONCLUSION(S)<br><br>As Above.

Representative edited model-generated reports (additions are in underlined magenta while deletions are shown as red strikethroughs). AP: anteroposterior; PA: posteroanterior.

© 2025 Huang J et al. *JAMA Network Open*.

eTable 6: Regression outputs for mixed-effects models

Fixed effects	Estimate	Standard error	z value
**Read Time**			
Intercept	170.35	35.91	4.74
Model Type	24.96	14.09	1.77
Study Type	37.72	9.40	4.02
Model:Study Type	8.83	11.06	0.80
**Clinical Accuracy Score**			
Model Type	0.30	0.20	1.52
Study Type	0.83	0.24	3.50
Model:Study Type	0.36	0.27	1.32
**Text Quality Score**			
Model Type	0.83	0.29	1.81
Study Type	1.78	0.47	2.43
Model:Study Type	0.81	0.47	1.71

Note that “Model:Study Type” denotes interaction effects.

© 2025 Huang J et al. *JAMA Network Open*.

eTable 7: Evaluation using automated metrics

Dataset	Radiograph type	Model	RadCliQ (v0)	RadGraph F1	CheXbert vector	BLEU-4
Internal held-out test dataset	Chest (n=5,000)	**AI model (ours)**	2.94 [2.88, 3.01]	0.254 [0.239, 0.268]	0.469 [0.453, 0.484]	0.218 [0.206, 0.229]
	Non-chest (n=5,000)	**AI model (ours)**	2.38† [2.33, 2.42]	0.193† [0.184, 0.202]	0.745† [0.733, 0.757]	0.176 [0.168, 0.183]
Prospective internal dataset	Chest (n=5,000)	**AI model (ours)**	3.24 [3.18, 3.31]	0.291 [0.274, 0.305]	0.454 [0.440, 0.469]	0.126 [0.115, 0.136]
	Non-chest (n=5,000)	**AI model (ours)**	3.13† [3.08, 3.18]	0.180† [0.173, 0.188]	0.731† [0.721, 0.742]	0.057 [0.053, 0.061]
MIMIC-CXR external dataset	Chest (n=3,269)	**AI model (ours, zero-shot)**	4.02 [3.98, 4.05]	0.163 [0.158, 0.168]	0.362 [0.350, 0.375]	0.021 [0.020, 0.023]
		Flamingo-CXR	--	0.205	--	0.101
		LLaVA-Rad	--	0.294	--	0.154
		MAIRA-1	3.10 [3.07, 3.14]	0.243 [0.237, 0.248]	0.440 [0.431, 0.449]	0.142 [0.137, 0.147]
		RayDINO	3.07 [3.04, 3.11]	0.239 [0.233, 0.246]	0.448 [0.440, 0.456]	0.138 [0.134, 0.142]
		**AI model (ours, finetuned)**	3.07 [3.03, 3.11]	0.229 [0.221, 0.239]	0.457 [0.445, 0.471]	0.116 [0.111, 0.120]
		MedVersa	2.74 [2.69, 2.79]	0.300 [0.291, 0.308]	0.466 [0.453, 0.468]	0.160 [0.153, 0.167]
		MAIRA-2 13B	2.59 [2.56, 2.63]	0.359 [0.356, 0.366]	0.513 [0.510, 0.521]	0.243 [0.237, 0.249]

The model was evaluated on 5,000 chest and non-chest studies each from the internal held-out test dataset and the prospective internal dataset using automated metrics of radiograph report quality. Evaluation was also performed on the MIMIC-CXR test set, a dataset of chest radiographs commonly used for model development and validation. Comparisons to recently published models are presented. Note that CheXbert, RadGraph, and RadCliQ were developed specifically for chest radiograph report evaluation.

† Indicates metric developed and validated for chest radiographs only.

© 2025 Huang J et al. *JAMA Network Open*.

eReferences

1. Vaswani A, Shazeer N, Parmar N, et al. Attention is All you Need. NeurIPS 2017: 5998-6008.
2. Dosovitskiy A, Beyer L, Kolesnikov A, et al. An Image is Worth 16x16 Words: Transformers for Image Recognition at Scale. ICLR 2021.
3. Caron M, Touvron H, Misra I, et al. Emerging Properties in Self-Supervised Vision Transformers. ICCV 2021: 9630-9640.
4. Zhang S, Roller S, Goyal N, et al. OPT: Open Pre-trained Transformer Language Models. *arXiv.* Preprint posted online June 21, 2022.
5. Liu Y, Ott M, Goyal N, et al. RoBERTa: A Robustly Optimized BERT Pretraining Approach. *arXiv.* Preprint posted online July 26, 2019.
6. Sennrich R, Haddow B, Birch A. Neural Machine Translation of Rare Words with Subword Units. ACL 2016: 1715-1725.
7. He K, Zhang X, Ren S, Sun J. Delving Deep into Rectifiers: Surpassing Human-Level Performance on ImageNet Classification. ICCV 2015: 1026-1034.
8. Smit A, Jain S, Rajpurkar P, Pareek A, Ng AY, Lungren MP. Combining Automatic Labelers and Expert Annotations for Accurate Radiology Report Labeling Using BERT. EMNLP 2020: 1500-1519.
9. Yu F, Endo M, Krishnan R, et al. Evaluating progress in automatic chest X-ray radiology report generation. *Patterns.* 2023;4(9).
10. Johnson AEW, Pollard TJ, Berkowitz SJ, et al. MIMIC-CXR, a de-identified publicly available database of chest radiographs with free-text reports. *Scientific Data.* 2019;6(1):317.
11. Jain S, Agrawal A, Saporta A, et al. RadGraph: Extracting Clinical Entities and Relations from Radiology Reports. NeurIPS Datasets and Benchmarks 2021.
12. Meister C, Pimentel T, Wiher G, Cotterell R. Locally Typical Sampling. *Trans Assoc Comput Linguistics.* 2023;11:102-121.
13. Huang J, Neill L, Wittbrodt M, et al. Generative Artificial Intelligence for Chest Radiograph Interpretation in the Emergency Department. *JAMA Network Open.* 2023;6(10):e2336100-e2336100.
14. Jackson VP, Cushing T, Abujudeh HH, et al. RADPEER Scoring White Paper. *Journal of the American College of Radiology.* 2009;6(1):21-25.
15. Tiu E, Talius E, Patel P, Langlotz CP, Ng AY, Rajpurkar P. Expert-level detection of pathologies from unannotated chest X-ray images via self-supervised learning. *Nature Biomedical Engineering.* 2022;6(12):1399-1406.
16. Hyland SL, Bannur S, Bouzid K, et al. MAIRA-1: A specialised large multimodal model for radiology report generation. *arXiv.* Preprint posted online April 26, 2024.
17. Bannur S, Bouzid K, Castro DC, et al. MAIRA-2: Grounded Radiology Report Generation. *arXiv.* Preprint posted online September 20, 2024.
18. Chaves JMZ, Huang S-C, Xu Y, et al. Towards a clinically accessible radiology foundation model: open-access and lightweight, with automated evaluation. *arXiv*. Preprint posted online June 27, 2024.
19. Zhou H-Y, Adithan S, Nicolás Acosta J, Topol EJ, Rajpurkar P. A Generalist Learner for Multifaceted Medical Image Interpretation. *arXiv*. Preprint posted online May 13, 2024.
20. Tanno R, Barrett DGT, Sellergren A, et al. Collaboration between clinicians and vision–language models in radiology report generation. *Nature Medicine.* 2025;31(2):599-608.

© 2025 Huang J et al. *JAMA Network Open*.

21. Moutakanni T, Bojanowski P, Chassagnon G, et al. Advancing human-centric AI for robust X-ray analysis through holistic self-supervised learning. *arXiv.* Preprint posted online May 2, 2024.

© 2025 Huang J et al. *JAMA Network Open*.
